# The landscape of antibody binding in SARS-CoV-2 infection

**DOI:** 10.1371/journal.pbio.3001265

**Published:** 2021-06-18

**Authors:** Anna S. Heffron, Sean J. McIlwain, Maya F. Amjadi, David A. Baker, Saniya Khullar, Tammy Armbrust, Peter J. Halfmann, Yoshihiro Kawaoka, Ajay K. Sethi, Ann C. Palmenberg, Miriam A. Shelef, David H. O’Connor, Irene M. Ong

**Affiliations:** 1 Department of Pathology and Laboratory Medicine, University of Wisconsin-Madison, Madison, Wisconsin, United States of America; 2 Department of Biostatistics and Medical Informatics, University of Wisconsin-Madison, Madison, Wisconsin, United States of America; 3 University of Wisconsin Carbone Comprehensive Cancer Center, University of Wisconsin-Madison, Madison, Wisconsin, United States of America; 4 Department of Medicine, University of Wisconsin-Madison, Madison, Wisconsin, United States of America; 5 Department of Pathobiological Sciences, School of Veterinary Medicine, Influenza Research Institute, University of Wisconsin-Madison, Madison, Wisconsin, United States of America; 6 Department of Population Health Sciences, University of Wisconsin-Madison, Madison, Wisconsin, United States of America; 7 Department of Biochemistry, Institute for Molecular Virology, University of Wisconsin–Madison, Madison, Wisconsin, United States of America; 8 William S. Middleton Memorial Veterans Hospital, Madison, Wisconsin, United States of America; 9 Wisconsin National Primate Research Center, University of Wisconsin-Madison, Madison, Wisconsin, United States of America; 10 Department of Obstetrics and Gynecology, University of Wisconsin-Madison, Madison, Wisconsin, United States of America; 11 Center for Human Genomics and Precision Medicine, University of Wisconsin-Madison, Madison, Wisconsin, United States of America; Massachusetts General Hospital, UNITED STATES

## Abstract

The search for potential antibody-based diagnostics, vaccines, and therapeutics for pandemic severe acute respiratory syndrome coronavirus 2 (SARS-CoV-2) has focused almost exclusively on the spike (S) and nucleocapsid (N) proteins. Coronavirus membrane (M), ORF3a, and ORF8 proteins are humoral immunogens in other coronaviruses (CoVs) but remain largely uninvestigated for SARS-CoV-2. Here, we use ultradense peptide microarray mapping to show that SARS-CoV-2 infection induces robust antibody responses to epitopes throughout the SARS-CoV-2 proteome, particularly in M, in which 1 epitope achieved excellent diagnostic accuracy. We map 79 B cell epitopes throughout the SARS-CoV-2 proteome and demonstrate that antibodies that develop in response to SARS-CoV-2 infection bind homologous peptide sequences in the 6 other known human CoVs. We also confirm reactivity against 4 of our top-ranking epitopes by enzyme-linked immunosorbent assay (ELISA). Illness severity correlated with increased reactivity to 9 SARS-CoV-2 epitopes in S, M, N, and ORF3a in our population. Our results demonstrate previously unknown, highly reactive B cell epitopes throughout the full proteome of SARS-CoV-2 and other CoV proteins.

## Abstract 2

Profiling of antibody binding from naïve and COVID-19 convalescent human sera to the entire proteome of SARS-CoV-2 and other human, bat and pangolin coronaviruses identifies 79 B cell epitopes throughout the SARS-CoV-2 proteome, finding that the most sensitive and specific binding occurred in the membrane (M) protein, and revealing cross-reactivity patterns.

## Introduction

Antibodies correlate with protection from coronaviruses (CoVs) including severe acute respiratory syndrome coronavirus 2 (SARS-CoV-2) [1–8], severe acute respiratory syndrome coronavirus (SARS-CoV) [8–12], and Middle Eastern respiratory syndrome coronavirus (MERS-CoV) [8,13–16]. All CoVs encode 4 main structural proteins, spike (S), envelope (E), membrane (M), and nucleocapsid (N), as well as multiple nonstructural proteins and accessory proteins [17]. In SARS-CoV-2, anti-S and anti-N antibodies have received the most attention to date [1–8], including in serology-based diagnostic tests [1–5] and vaccine candidates [6–8]. The immunogenicity of S-based vaccines is variable [18,19], so better representation of the breadth of antibody reactivity in vaccines, therapeutics, and diagnostics will be important as the pandemic continues especially as new variants emerge. Prior reports observed that not all individuals infected with SARS-CoV-2 produce detectable antibodies against S or N [1–5], indicating a need for expanded antibody-based options.

Much less is known about antibody responses to other SARS-CoV-2 proteins, although data from other CoVs suggest they may be important. Antibodies against SARS-CoV M can be more potent than antibodies against SARS-CoV S [20–22], and some experimental SARS-CoV and MERS-CoV vaccines elicit responses to M, E, and ORF8 [8]. Additionally, previous work has demonstrated humoral cross-reactivity between CoVs [7,11,23–26] and suggested it could be protective [26,27], although full-proteome cross-reactivity has not been investigated.

We designed a peptide microarray tiling the proteomes of SARS-CoV-2 and 8 other human and animal CoVs in order to assess antibody epitope specificity and potential cross-reactivity with other CoVs. We examined immunoglobulin G (IgG) antibody responses in 40 coronavirus disease 2019 (COVID-19) convalescent patients and 20 SARS-CoV-2-naïve controls. Independent enzyme-linked immunosorbent assays (ELISAs) confirm 4 of the highest-performing epitopes. We detected antibody responses to epitopes throughout the SARS-CoV-2 proteome, with several antibodies exhibiting apparent cross-reactive binding to homologous epitopes in multiple other CoVs.

## Results

### SARS-CoV-2-naïve controls show consistent binding in “common cold” CoVs and limited binding in SARS-CoV-2, SARS-CoV, and MERS-CoV

**Fig 1:**
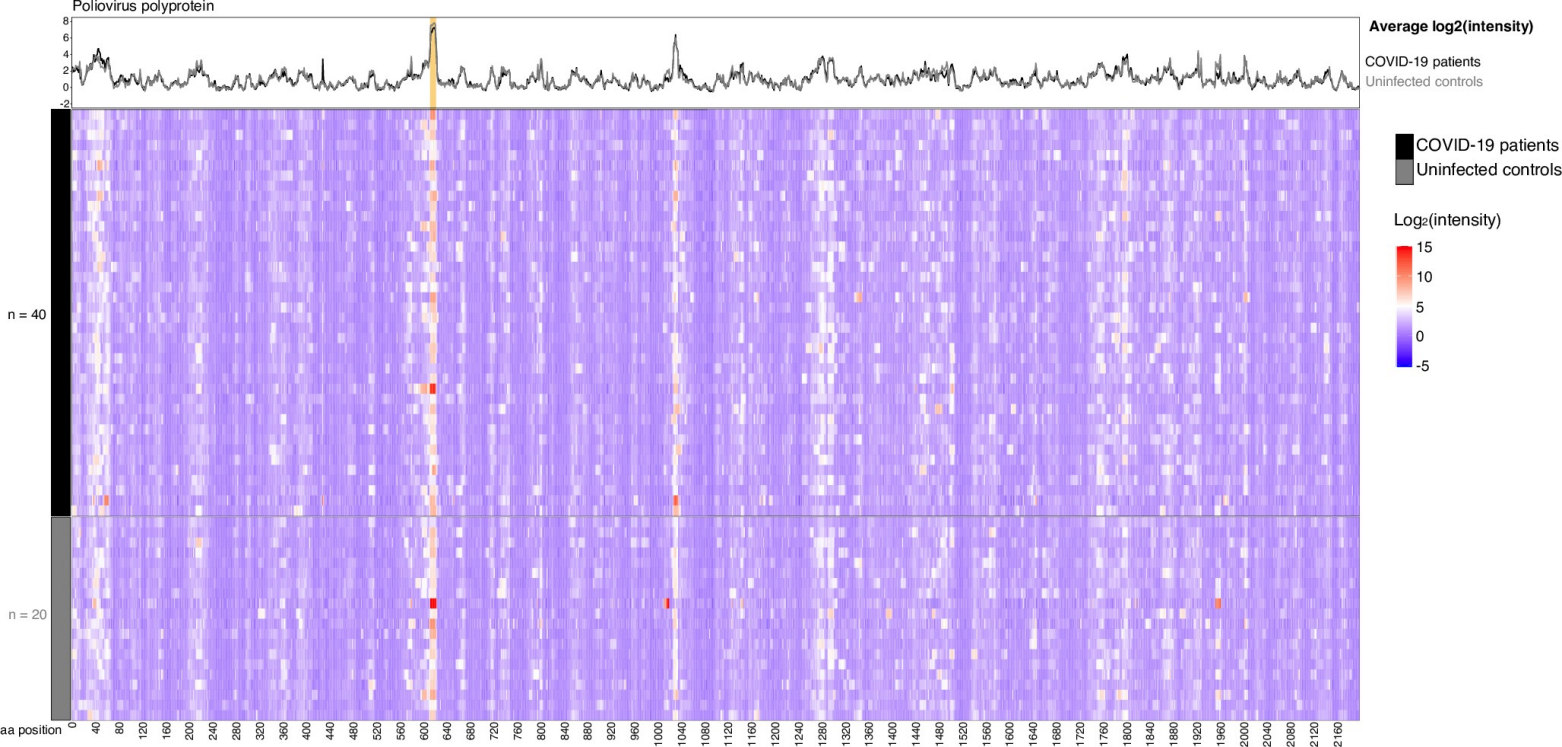
Patients and controls show reactivity to a poliovirus control. Sera from 20 controls collected before 2019 were assayed for IgG binding to the full proteome of human poliovirus 1 on a peptide microarray. Binding was measured as reactivity that was >3.00 standard deviations above the mean for the log_2_-quantile normalized array data. Patients and controls alike showed reactivity to a well-documented linear poliovirus epitope (start position 613 [IEDB.org]; orange shading in line plot). The data used in this analysis can be accessed online at: https://github.com/Ong-Research/UW_Adult_Covid-19. COVID-19, coronavirus disease 2019; IgG, immunoglobulin G.

**Fig 2:**
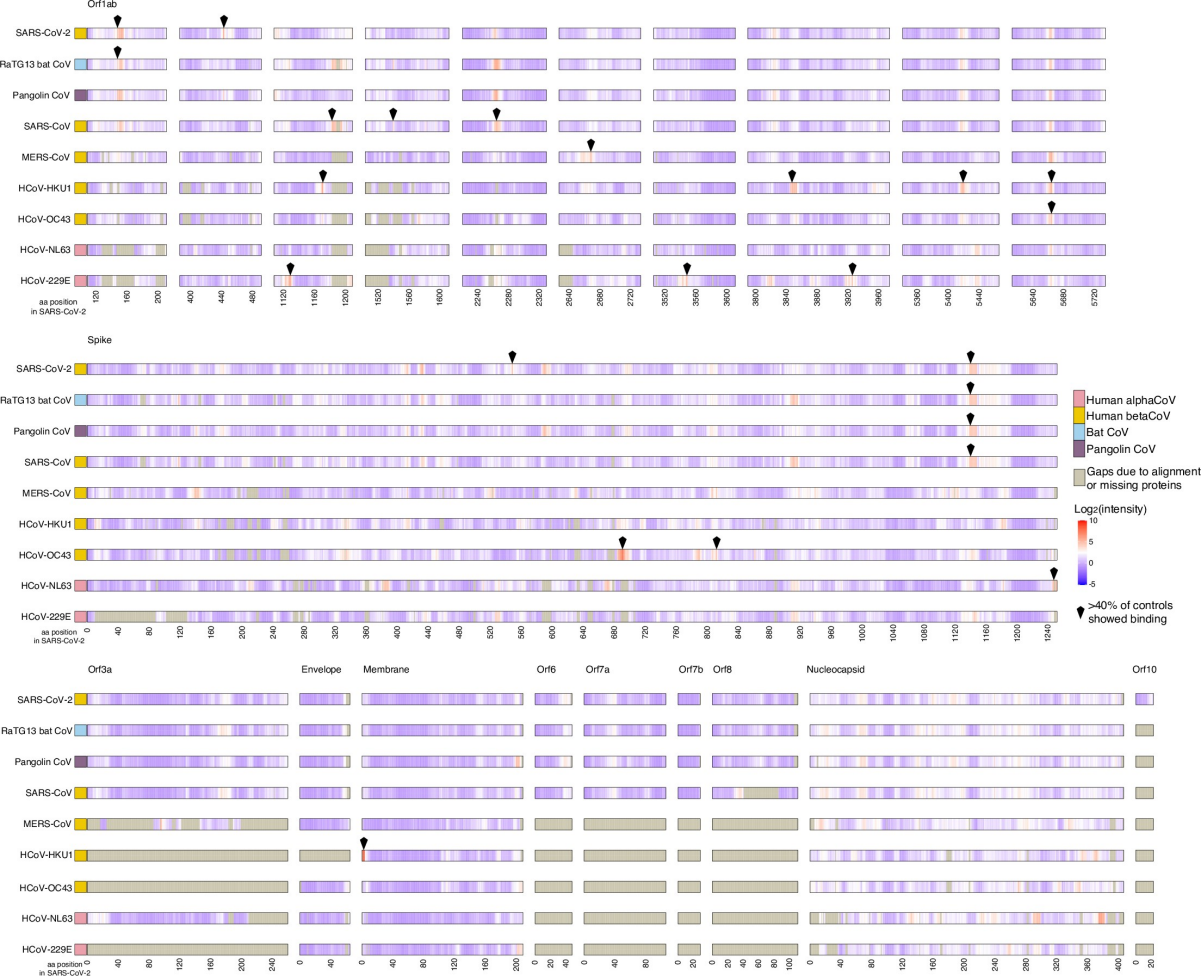
Control sera show reactivity to CCCoVs and to SARS-CoV, MERS-CoV, and SARS-CoV-2. Sera from 20 controls collected before 2019 were assayed for IgG binding to the full proteomes of 9 CoVs on a peptide microarray. Viral proteins are shown aligned to the SARS-CoV-2 proteome with each virus having an individual panel; SARS-CoV-2 aa position is represented on the x-axis. Binding was measured as reactivity that was >3.00 standard deviations above the mean for the log_2_-quantile normalized array data. Peptides for which >40% of the controls showed binding are indicated by a black diamond. The data used in this analysis can be accessed online at: https://github.com/Ong-Research/UW_Adult_Covid-19. aa, amino acid; CCCoVs, “common cold” CoVs; CoV, coronavirus; IgG, immunoglobulin G; MERS-CoV, Middle Eastern respiratory syndrome coronavirus; SARS-CoV, severe acute respiratory syndrome coronavirus; SARS-CoV-2, severe acute respiratory syndrome coronavirus 2.

Greater than 90% of adult humans are seropositive for the human “common cold” CoVs (CCCoVs: HCoV-HKU1, HCoV-OC43, HCoV-NL63, and HCoV-229E) [28,29], but the effect of these preexisting antibodies upon immune responses to SARS-CoV-2 or other CoVs remains uncertain. We measured IgG reactivity in sera from 20 SARS-CoV-2-naïve controls to CoV linear peptides, considering reactivity that was >3.00 standard deviations above the mean for the log_2_-quantile normalized array data to be indicative of antibody binding [30]. All sera (SARS-CoV-2-naïve and COVID-19-convalescent) exhibited binding in known epitopes of at least one of the control non-CoV strains (poliovirus vaccine and rhinovirus; Fig 1, S1 Data), and all were collected in Wisconsin, United States of America, where exposure to SARS-CoV or MERS-CoV was extremely unlikely. We found that at least 1 epitope in structural or accessory proteins showed binding in 100% of controls for HCoV-HKU1, 85% of controls for HCoV-OC43, 65% for HCoV-NL63, and 55% for HCoV-229E (Fig 2, S2 Data). The apparent cross-reactive binding was observed in 45% of controls for MERS-CoV, 50% for SARS-CoV, and 50% for SARS-CoV-2 (S2 Data). We completed neutralization assays on 12 of these control samples and on 18 additional samples from other SARS-CoV-2-naïve controls collected before 2019, and none of these had detectable neutralization activity against SARS-CoV-2 [31] (S1 Table, S1 Fig).

### SARS-CoV-2 infection induces antibodies binding throughout the proteome

We aimed to map the full breadth of IgG binding induced by SARS-CoV-2 infection and to rank the identified epitopes in terms of likelihood of immunodominance. We defined epitope recognition as antibody binding to contiguous peptides in which the average log_2_-normalized intensity for patients was at least 2-fold greater than for controls with *t* test statistics yielding adjusted *p*-values <0.1. We chose these criteria, rather than the 3.00 standard deviation cutoff (S2 Data), in order to ensure that binding detected would be greater than background binding seen in controls (2-fold greater) and to remove regions of binding that were not at least weakly significantly different from controls (adjusted *p* < 0.1). All COVID-19 convalescent patients’ sera bound multiple epitopes in SARS-CoV-2, including in 2 patients who did not have detectable neutralizing antibodies in neutralization assays (S1 Table, S1 Fig). Top-ranking epitopes had greater correlations (0.7 and greater) with neutralization titers (Table 1).

**Table 1:** Profiling antibody binding in 40 COVID-19 convalescent patients compared to 20 naïve controls identifies B cell epitopes in SARS-CoV-2 (all data is log_2_-normalized).

Protein	First aa position	Epitope identifier	Sequence	Minimum *p*-value	Minimum signal	Maximum signal	Mean signal	Mean fold change	Shown to be neutralizing (PMID)	Bioinformatically predicted (PMID)	Pearson correlation of binding with neutralization titer
**M**	1	1-M-24	MADSNGTITVEELKKLLEQWNLVI	2.19E-23	3.45	5.97	4.45	4.77		32183941	0.77
**N**	384	384-N-33	QRQKKQQTVTLLPAADLDDFSKQLQQSMSSADS	9.10E-18	2.63	5.63	4.48	3.87		32183941	0.73
**S**	568	568-S-26	DIADTTDAVRDPQTLEILDITPCSFG	1.61E-14	0.64	4.44	3.55	4.83		32843695	0.77
**S**	1247	1247-S-27	CCSCGSCCKFDEDDSEPVLKGVKLHYT	7.33E-13	2.06	4.98	3.77	3.96		32183941	0.69
**N**	208	208-N-31	ARMAGNGGDAALALLLLDRLNQLESKMSGKG	1.15E-12	0.95	3.93	2.45	2.60		32183941	0.76
**S**	807	807-S-26	PDPSKPSKRSFIEDLLFNKVTLADAG	4.68E-12	3.75	6.29	5.33	3.62	32483236	32183941	0.69
**S**	553	553-S-26	TESNKKFLPFQQFGRDIADTTDAVRD	7.22E-12	2.01	5.79	4.41	3.66	32483236; 32612199; 32895485	32183941; 32843695	0.81
**S**	785	785-S-27	VKQIYKTPPIKDFGGFNFSQILPDPSK	3.42E-11	2.22	4.95	3.80	2.99		32183941; 32843695	0.67
**S**	1140	1140-S-25	PLQPELDSFKEELDKYFKNHTSPDV	1.08E-09	3.40	6.71	5.84	3.16	32612199	32183941; 32843695	0.63
**S**	624	624-S-23	IHADQLTPTWRVYSTGSNVFQTR	8.75E-09	0.84	2.25	1.50	1.98	32612199	32183941	0.70
**M**	181	181-M-32	LGASQRVAGDSGFAAYSRYRIGNYKLNTDHSS	1.35E-08	1.44	3.86	2.34	2.61		32183941	0.66
**N**	28	28-N-28	QNGERSGARSKQRRPQGLPNNTASWFTA	1.78E-08	1.75	4.13	2.94	1.86		32183941	0.52
**ORF1ab**	4514	4514-ORF1ab-16	YTMADLVYALRHFDEG	3.44E-08	2.88	2.88	2.88	3.84			0.76
**M**	152	152-M-26	AGHHLGRCDIKDLPKEITVATSRTLS	6.33E-08	2.96	4.26	3.44	2.96		32183941	0.60
**S**	549	549-S-18	TGVLTESNKKFLPFQQFG	1.14E-07	4.35	4.96	4.72	3.00		32183941	0.73
**S**	685	685-S-25	RSVASQSIIAYTMSLGAENSVAYSN	3.26E-07	1.18	2.95	2.28	2.16		32183941	0.73
**N**	249	249-N-18	KSAAEASKKPRQKRTATK	2.03E-06	2.10	3.39	2.72	1.60		32183941	0.51
**M**	205	205-M-18	KLNTDHSSSSDNIALLVQ	2.64E-06	2.14	3.32	2.41	2.93		32183941	0.65
**ORF1ab**	5999	5999-ORF1ab-16	ITREEAIRHVRAWIGF	4.02E-06	1.56	1.56	1.56	2.17			0.66
**ORF1ab**	1239	1239-ORF1ab-18	VTTTLEETKFLTENLLLY	6.69E-06	1.38	1.55	1.49	1.60			0.45
**ORF1ab**	2309	2309-ORF1ab-16	ITISSFKWDLTAFGLV	6.79E-06	1.05	1.05	1.05	2.08			0.61
**S**	613	613-S-25	QDVNCTEVPVAIHADQLTPTWRVYS	8.65E-06	1.52	2.90	2.35	2.12		32183941	0.66
**ORF1ab**	1551	1551-ORF1ab-16	ITFDNLKTLLSLREVR	1.46E-05	1.01	1.01	1.01	1.75			0.65
**ORF1ab**	6057	6057-ORF1ab-17	DFSRVSAKPPPGDQFKH	6.02E-05	2.85	3.32	3.09	1.42			0.53
**N**	153	153-N-26	NNAAIVLQLPQGTTLPKGFYAEGSRG	7.56E-05	1.91	6.02	4.70	3.17		32183941	0.59
**ORF1ab**	1720	1720-ORF1ab-16	KTVGELGDVRETMSYL	9.05E-05	1.74	1.74	1.74	1.41			0.53
**S**	635	635-S-20	VYSTGSNVFQTRAGCLIGAE	9.25E-05	0.98	1.91	1.35	1.33		32183941	0.53
**N**	14	14-N-17	RITFGGPSDSTGSNQNG	9.46E-05	3.35	3.82	3.59	2.16			0.51
**N**	7	7-N-21	QNQRNAPRITFGGPSDSTGSN	1.70E-04	3.24	3.70	3.39	2.34		32183941	0.56
**S**	940	940-S-16	STASALGKLQDVVNQN	1.75E-04	2.53	2.53	2.53	2.05			0.57
**S**	1155	1155-S-20	YFKNHTSPDVDLGDISGINA	2.16E-04	2.44	4.07	3.27	2.16		32183941	0.66
**N**	338	338-N-19	KLDDKDPNFKDQVILLNKH	3.62E-04	1.89	2.45	2.22	1.76			0.56
**S**	404	404-S-18	GDEVRQIAPGQTGKIADY	3.77E-04	2.00	2.72	2.36	1.58		32183941; 32843695	0.44
**ORF8**	60	60-ORF8-20	LCVDEAGSKSPIQYIDIGNY	4.24E-04	2.31	3.18	2.66	1.82			0.45
**N**	376	376-N-22	ADETQALPQRQKKQQTVTLLPA	5.98E-04	2.31	2.92	2.67	1.93		32183941	0.43
**ORF3a**	252	252-ORF3a-24	SSGVVNPVMEPIYDEPTTTTSVPL	7.03E-04	3.20	4.23	3.52	2.16			0.56
**N**	230	230-N-21	LESKMSGKGQQQQGQTVTKKS	8.70E-04	2.88	3.96	3.28	2.10		32183941	0.39
**N**	94	94-N-16	IRGGDGKMKDLSPRWY	1.33E-03	2.64	2.64	2.64	1.60		32183941	0.61
**N**	356	356-N-16	HIDAYKTFPPTEPKKD	2.06E-03	3.30	3.30	3.30	1.84			0.47
**S**	536	536-S-17	NKCVNFNFNGLTGTGVL	2.10E-03	1.81	1.95	1.88	1.15			0.45
**ORF8**	12	12-ORF8-16	TVAAFHQECSLQSCTQ	2.24E-03	1.03	1.03	1.03	1.00			0.41
**S**	798	798-S-17	GGFNFSQILPDPSKPSK	2.98E-03	2.75	3.17	2.96	1.38		32183941	0.39
**N**	227	227-N-17	LNQLESKMSGKGQQQQG	3.53E-03	3.39	3.51	3.45	1.94			0.32
**ORF8**	66	66-ORF8-18	GSKSPIQYIDIGNYTVSC	3.99E-03	2.00	2.39	2.20	1.52			0.37
**ORF1ab**	4451	4451-ORF1ab-16	KDEDDNLIDSYFVVKR	4.33E-03	0.97	0.97	0.97	1.19			0.32
**S**	289	289-S-17	VDCALDPLSETKCTLKS	5.49E-03	2.19	2.20	2.19	1.30		32183941	0.60
**N**	117	117-N-19	PEAGLPYGANKDGIIWVAT	5.73E-03	0.95	2.73	1.67	1.56			0.57
**M**	175	175-M-20	TLSYYKLGASQRVAGDSGFA	5.78E-03	1.88	2.65	2.35	1.30			0.39
**S**	644	644-S-16	QTRAGCLIGAEHVNNS	6.88E-03	1.66	1.66	1.66	1.22		32183941	0.47
**ORF6**	9	9-ORF6-16	VTIAEILLIIMRTFKV	6.98E-03	1.07	1.07	1.07	1.09			0.54
**N**	242	242-N-19	QGQTVTKKSAAEASKKPRQ	7.50E-03	2.98	3.15	3.09	1.42		32183941	0.46
**S**	656	656-S-17	VNNSYECDIPIGAGICA	8.91E-03	3.32	3.36	3.34	1.79		32183941	0.62
**S**	541	541-S-16	FNFNGLTGTGVLTESN	9.09E-03	1.68	1.68	1.68	1.36			0.46
**S**	844	844-S-16	IAARDLICAQKFNGLT	9.63E-03	1.93	1.93	1.93	1.19			0.64
**S**	804	804-S-17	QILPDPSKPSKRSFIED	1.42E-02	2.61	2.97	2.79	1.45		32183941	0.28
**N**	126	126-N-17	NKDGIIWVATEGALNTP	1.43E-02	1.16	1.40	1.28	1.11			0.43
**N**	96	96-N-17	GGDGKMKDLSPRWYFYY	1.49E-02	0.85	1.41	1.13	1.19		32183941	0.46
**ORF3a**	235	235-ORF3a-17	KIVDEPEEHVQIHTIDG	1.56E-02	1.20	1.20	1.20	1.46			0.27
**N**	122	122-N-16	PYGANKDGIIWVATEG	1.95E-02	0.85	0.85	0.85	1.12			0.54
**ORF8**	53	53-ORF8-16	KSAPLIELCVDEAGSK	1.95E-02	1.43	1.43	1.43	1.03			0.08
**N**	124	124-N-16	GANKDGIIWVATEGAL	2.55E-02	0.91	0.91	0.91	1.03			0.47
**N**	336	336-N-16	AIKLDDKDPNFKDQVI	2.58E-02	3.31	3.31	3.31	1.43			0.43
**ORF1ab**	1546	1546-ORF1ab-16	LDGEVITFDNLKTLLS	3.75E-02	0.94	0.94	0.94	1.07			0.20
**S**	306	306-S-16	FTVEKGIYQTSNFRVQ	4.00E-02	1.92	1.92	1.92	1.38		32183941	0.46
**S**	241	241-S-16	LLALHRSYLTPGDSSS	4.06E-02	1.12	1.12	1.12	1.06		32183941	0.47
**S**	768	768-S-18	TGIAVEQDKNTQEVFAQV	4.33E-02	3.23	4.15	3.76	2.16		32183941	0.25
**ORF3a**	16	16-ORF3a-16	KQGEIKDATPSDFVRA	4.40E-02	3.40	3.40	3.40	1.87			0.43
**S**	1164	1164-S-16	VDLGDISGINASVVNI	5.04E-02	3.29	3.29	3.29	1.55		32183941	0.53
**S**	172	172-S-16	SQPFLMDLEGKQGNFK	5.10E-02	2.69	2.69	2.69	1.09		32183941	0.37
**ORF3a**	21	21-ORF3a-16	KDATPSDFVRATATIP	5.25E-02	2.03	2.03	2.03	1.43			0.40
**ORF1ab**	2584	2584-ORF1ab-16	AEVAVKMFDAYVNTFS	6.57E-02	1.54	1.54	1.54	1.04			0.20
**S**	1178	1178-S-16	NIQKEIDRLNEVAKNL	7.05E-02	4.19	4.19	4.19	1.63			0.60
**S**	661	661-S-16	ECDIPIGAGICASYQT	7.17E-02	2.35	2.35	2.35	1.32		32183941	0.56
**ORF3a**	18	18-ORF3a-16	GEIKDATPSDFVRATA	7.68E-02	2.57	2.57	2.57	1.66			0.33
**S**	410	410-S-16	IAPGQTGKIADYNYKL	7.72E-02	3.23	3.23	3.23	1.35		32183941; 32843695	0.33
**S**	1161	1161-S-17	SPDVDLGDISGINASVV	7.84E-02	3.88	4.49	4.18	1.75		32183941	0.50
**S**	761	761-S-16	TQLNRALTGIAVEQDK	8.12E-02	3.34	3.34	3.34	2.10		32183941	0.24
**ORF1ab**	1681	1681-ORF1ab-16	LTLQQIELKFNPPALQ	8.28E-02	1.64	1.64	1.64	1.19			0.56
**ORF1ab**	1572	1572-ORF1ab-16	TTVDNINLHTQVVDMS	8.60E-02	2.28	2.28	2.28	1.40			0.32

aa, amino acid; COVID-19, coronavirus disease 2019; M, membrane; N, nucleocapsid; S, spike; SARS-CoV-2, severe acute respiratory syndrome coronavirus 2.

**Fig 3:**
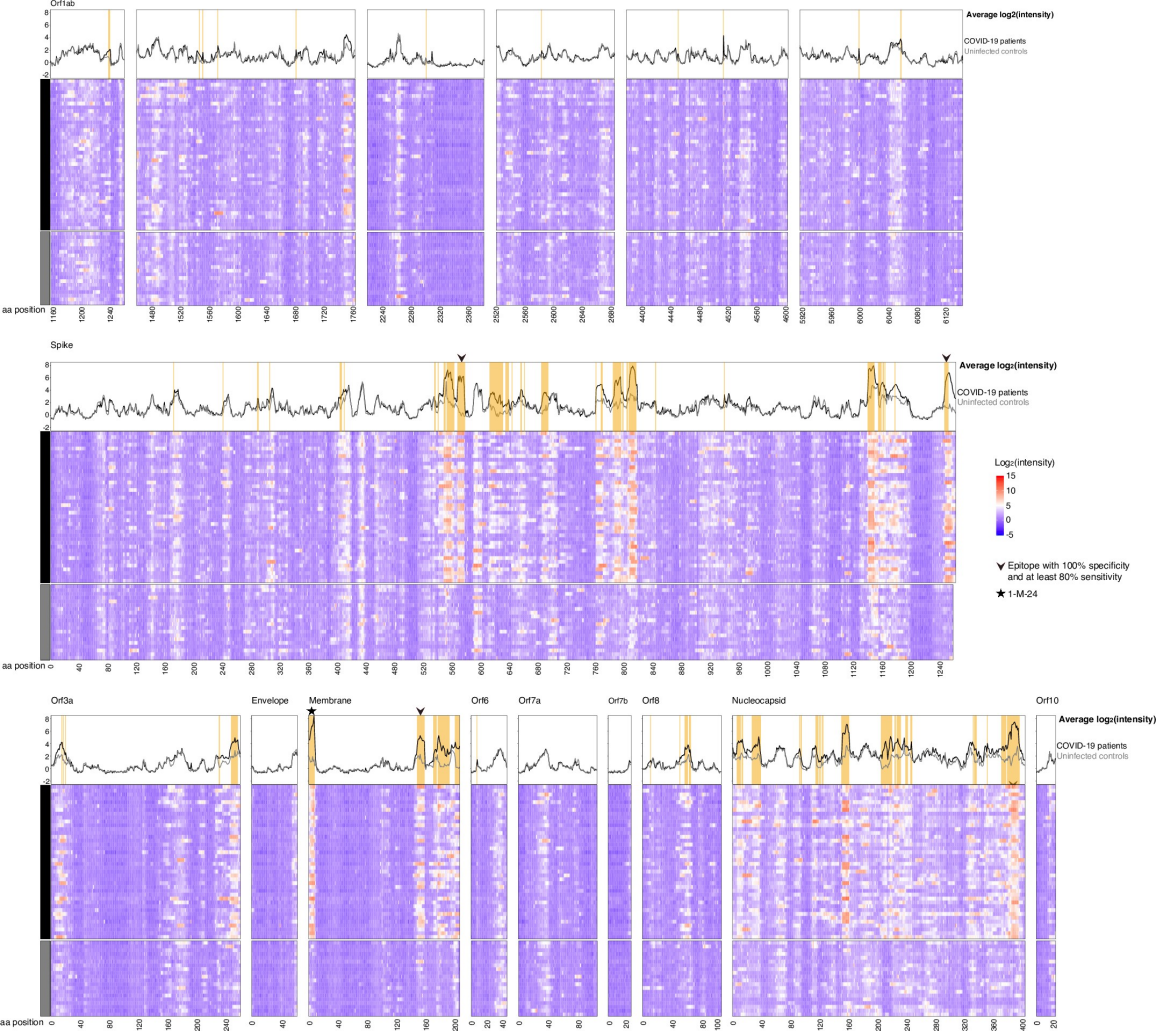
Anti-SARS-CoV-2 antibodies bind throughout the viral proteome. Sera from 40 COVID-19 convalescent patients were assayed for IgG binding to the full SARS-CoV-2 proteome on a peptide microarray. B cell epitopes were defined as peptides in which patients’ average log_2_-normalized intensity (black lines in line plots) is 2-fold greater than controls’ (gray lines in line plots), and *t* test statistics yield adjusted *p*-values <0.1; epitopes are identified by orange shading in the line plots. Epitopes having at least 100% specificity and at least 80% sensitivity for SARS-CoV-2 infection are indicated by a black arrow. The 1-M-24 epitope, which had the highest combined reactivity, specificity, and sensitivity of all epitopes we defined, is indicated by a black star. The data used in this analysis can be accessed online at: https://github.com/Ong-Research/UW_Adult_Covid-19. aa, amino acid; COVID-19, coronavirus disease 2019; IgG, immunoglobulin G; SARS-CoV-2, severe acute respiratory syndrome coronavirus 2.

**Fig 4:**
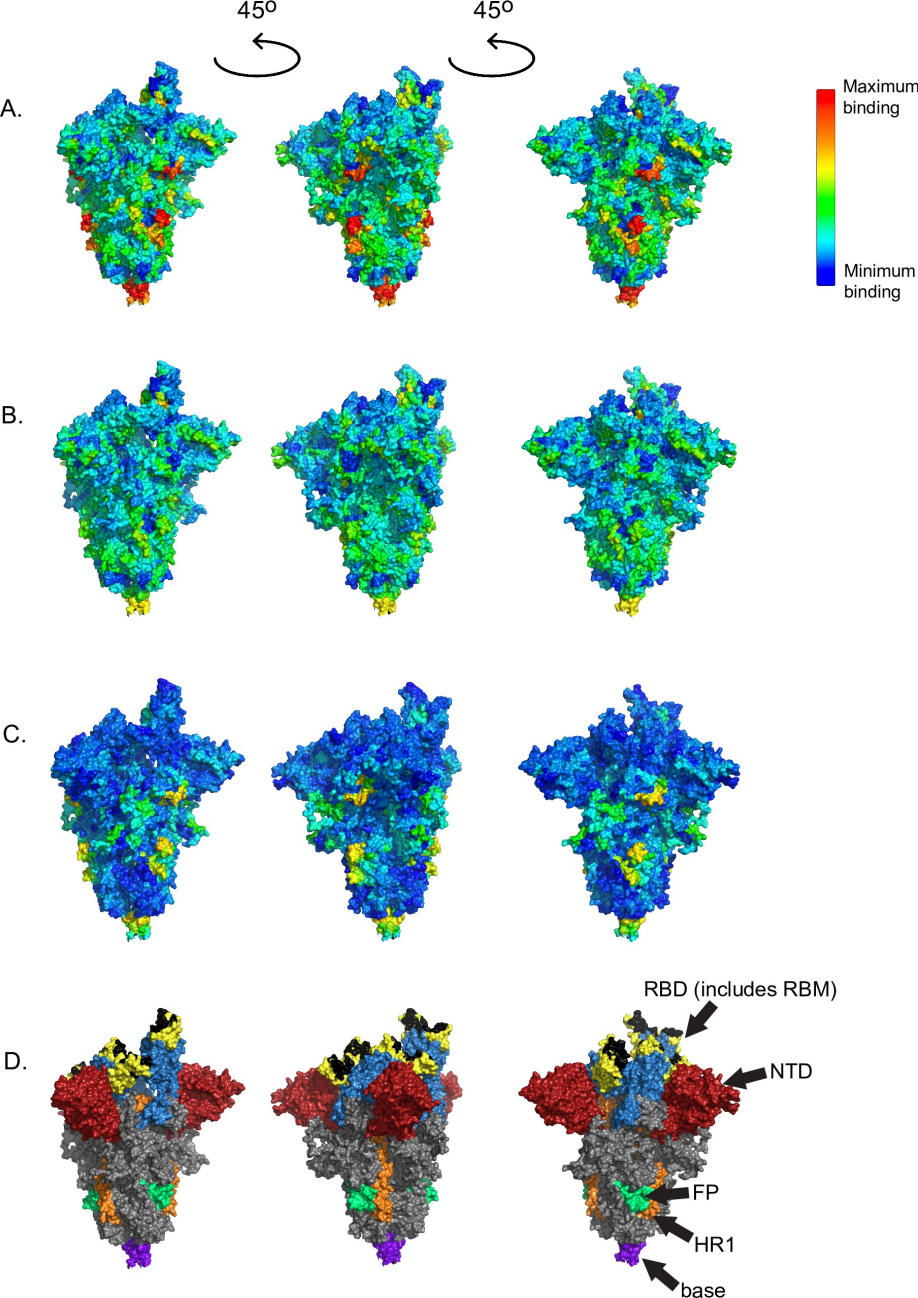
Anti-SARS-CoV-2 antibodies to S protein show the highest binding in the fusion cleavage site. Binding reactivities were localized on a coordinate file for a trimer of the SARS-CoV-2 S protein using a dark blue (low, 0.00 fluorescence intensity) to red (high, 9.00 fluorescence intensity) color scale. (A) COVID-19 convalescent patients, (B) naïve controls, and (C) the difference between patients and controls are shown. The highest reactivity occurred in the fusion peptide (aa 788–806) and at the base of the extracellular portion of the molecule (aa 984–1163), with lower reactivity in the receptor-binding domain (aa 319–541). (D) Key regions of the S protein are labeled and colored. In the S1 subunit (aa 14–685), the NTD (aa 14–305) is red, and the RBD (aa 319–541) is blue. Within the RBD, the RBM (aa 438–506) is yellow, and the residues that bind to the ACE2 receptor (aa 446, 449, 453, 455–456, 473, 475–476, 484, 486–487, 489–490, 493, 496, 498, 500–502, and 505) are in black. In the S2 subunit (aa 686–1273), the FP (aa 788–806) is green, the HR1 (aa 912–984) is orange, and the base of the extracellular part of the protein (base, roughly aa 1140–1160) is purple [6,38]. The remainder of the protein is gray. The data used in this analysis can be accessed online at: https://github.com/Ong-Research/UW_Adult_Covid-19. AA, amino acid; ACE2, angiotensin converting enzyme 2; COVID-19, coronavirus disease 2019; FP, fusion peptide; HR1, heptad repeat 1; NTD, N-terminal domain; RBD, receptor-binding domain; RBM, receptor-binding motif; S, spike; SARS-CoV-2, severe acute respiratory syndrome coronavirus 2.

These criteria identified 79 B cell epitopes (Fig 3, Table 1) in S, M, N, ORF1ab, ORF3a, ORF6, and ORF8. We ranked these epitopes by minimum adjusted *p*-value for any 16-mer in the epitope in order to determine the greatest likelihood of difference from controls as a proxy for likelihood of immunodominance. The highest-ranking epitope occurred in the N-terminus of M (1-M-24). Patient sera showed high-magnitude reactivity (up to an average of 6.7 fluorescence intensity units) in other epitopes in S, M, N, and ORF3a, with lower-magnitude reactivity (average of <3.3 fluorescence intensity units) epitopes in other proteins. The epitopes with the greatest reactivity in S were located in the S2 subunit of the protein (residues 686–1273) rather than the S1 subunit (residues 14–685) [6] (Fig 3). The greatest reactivity in S occurred in the fusion peptide (residues 788–806) and at the base of the extracellular portion of the protein (between the heptad repeat 1 and heptad repeat 2, roughly residues 984–1163) (Figs 3 and 4). The highest magnitude antibody binding (red sites in Fig 4A) on S are below the flexible head region that must be in the “up” position for angiotensin converting enzyme 2 (ACE2) binding to occur. Notably less reactivity occurred in the receptor-binding domain (RBD) (residues 319–541) [6]. Four detected epitopes (553-S-26, 624-S-23, 807-S-26, and 1140-S-25) have previously been shown to be potently neutralizing [32–34], and all 4 of these were ranked within the top 10 of our 79 epitopes. Forty-two of our detected epitopes (including 1-M-24, 553-S-26, 624-S-23, 807-S-26, and 1140-S-25; Table 1) confirm bioinformatic predictions of antigenicity based on SARS-CoV and MERS-CoV [7,8,35–37], including each of the 12 top-ranking epitopes.

The highest specificity (100%) and sensitivity (98%), determined by linear discriminant analysis leave-one-out cross-validation, for any individual peptide was observed for a 16-mer within the 1-M-24 epitope: ITVEELKKLLEQWNLV (S2 Table). Fifteen additional individual peptides in M, S, and N had 100% measured specificity and at least 80% sensitivity (Table 2). Combinations of 1-M-24 with 1 of 5 other epitopes (384-N-33, 807-S-26, 6057-ORF1ab-17, 227-N-17, and 4451-ORF1ab-16) yielded an area under the curve receiver operating characteristic of 1.00 (S3 Table) based on linear discriminant analysis leave-one-out-cross-validation.

**Table 2:** Sixteen peptides in the SARS-CoV-2 proteome had 100% specificity and at least 80% sensitivity for SARS-CoV-2 infection in 40 COVID-19 convalescent patients compared to 20 naïve controls.

Protein	First aa position	Sequence	Specificity	Sensitivity	F1
M	8	ITVEELKKLLEQWNLV	1	0.98	0.99
M	7	TITVEELKKLLEQWNL	1	0.95	0.97
N	390	QTVTLLPAADLDDFSK	1	0.95	0.97
N	388	KQQTVTLLPAADLDDF	1	0.90	0.95
N	391	TVTLLPAADLDDFSKQ	1	0.90	0.95
S	570	ADTTDAVRDPQTLEIL	1	0.88	0.93
S	571	DTTDAVRDPQTLEILD	1	0.88	0.93
S	574	DAVRDPQTLEILDITP	1	0.85	0.92
S	576	VRDPQTLEILDITPCS	1	0.85	0.92
S	1253	CCKFDEDDSEPVLKGV	1	0.85	0.92
S	572	TTDAVRDPQTLEILDI	1	0.83	0.90
S	573	TDAVRDPQTLEILDIT	1	0.83	0.90
S	577	RDPQTLEILDITPCSF	1	0.83	0.90
S	1252	SCCKFDEDDSEPVLKG	1	0.83	0.90
M	162	KDLPKEITVATSRTLS	1	0.83	0.90
S	1250	CGSCCKFDEDDSEPVL	1	0.80	0.89

aa, amino acid; COVID-19, coronavirus disease 2019; M, membrane; N, nucleocapsid; S, spike; SARS-CoV-2, severe acute respiratory syndrome coronavirus 2.

### Anti-SARS-CoV-2 antibodies may cross-reactively bind peptides in other CoVs

We determined epitopes bound by anti-SARS-CoV-2 antibodies in non-SARS-CoV-2 CoVs by the same criteria we used to determine epitopes in SARS-CoV-2. Epitopes in these viruses were defined as binding by antibodies in COVID-19 convalescent sera to peptides at an average log_2_-normalized intensity at least 2-fold greater than in controls with *t* test statistics yielding adjusted *p*-values <0.1. Some of these epitopes were identical sequences with SARS-CoV-2, particularly in the RaTG13 bat betacoronavirus (β-CoV), the closest known relative of SARS-CoV-2 (96% nucleotide identity) [39,40], the pangolin CoV (85% nucleotide identity with SARS-CoV-2) [41], and SARS-CoV (78% identity) [39]. Cross-reactivity of an antibody is typically determined by evaluating a pure preparation of specific antibodies or by competition assays. However, since our Wisconsin controls are almost certainly naïve to MERS-CoV, SARS-CoV, and bat and pangolin CoVs, we can make predictions about cross-reactivity (as opposed to binding due to sequence identity).

**Fig 5:**
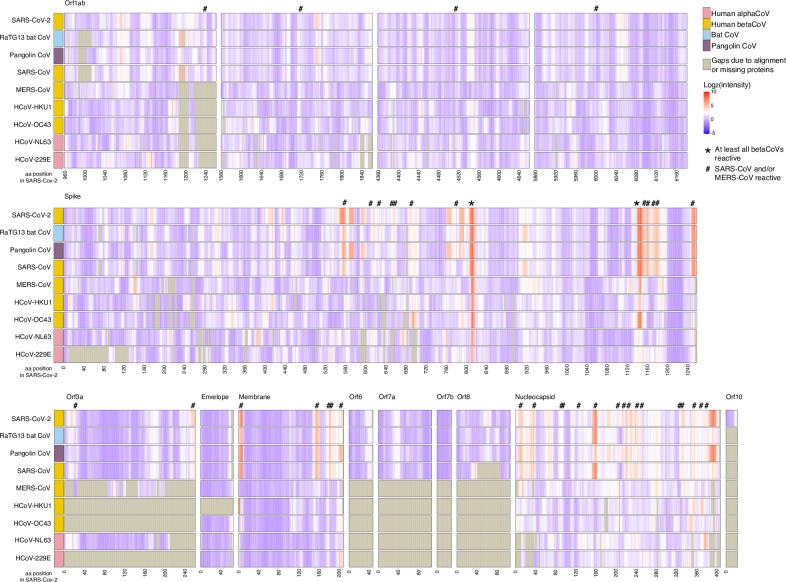
Anti-SARS-CoV-2 antibodies may cross-react with other CoVs. Sera from 40 COVID-19 convalescent patients were assayed for IgG binding to 9 CoVs on a peptide microarray; averages for all 40 are shown. Viral proteins are aligned to the SARS-CoV-2 proteome; SARS-CoV-2 aa position is represented on the x-axis. Regions that may be cross-reactive across all β-CoVs (*) or cross-reactive for SARS-CoV or MERS-CoV (#) are indicated. Gray shading indicates gaps due to alignment or lacking homologous proteins. Cross-reactive binding is defined as peptides in which patients’ average log_2_-normalized intensity is 2-fold greater than controls’ and *t* test statistics yield adjusted *p*-values <0.1. The data used in this analysis can be accessed online at: https://github.com/Ong-Research/UW_Adult_Covid-19. aa, amino acid, β-CoV, betacoronavirus; CoV, coronavirus; COVID-19, coronavirus disease 2019; IgG, immunoglobulin G; MERS-CoV, Middle Eastern respiratory syndrome coronavirus; SARS-CoV, severe acute respiratory syndrome coronavirus; SARS-CoV-2, severe acute respiratory syndrome coronavirus 2.

Antibodies in COVID-19-convalescent sera appeared to be cross-reactive with identical or homologous epitopes in S, M, N, ORF1ab, ORF3, ORF6, and ORF8 in other CoVs (Fig 5, S2 Fig, and S3 and S4 Data). Overall, the greatest number of epitopes in any non-SARS-CoV-2 CoV occurred in the RaTG13 bat β-CoV at 74 epitopes (60 identical to SARS-CoV-2, 13 homologous nonidentical, 1 without a homologous SARS-CoV-2 epitope). The second greatest number, 60 epitopes, occurred in the pangolin CoV (23 identical to SARS-CoV-2, 30 homologous nonidentical, 6 without a homologous SARS-CoV-2 epitope, 1 without a homologous region in SARS-CoV-2), and third SARS-CoV with 45 epitopes, (10 identical to SARS-CoV-2, 32 homologous nonidentical, 3 without a homologous SARS-CoV-2 epitope) (S3 and S4 Data). Most (8 of 12) of the epitopes that were not in areas having epitopes in the homologous SARS-CoV-2 region occurred in ORF1ab, with the others occurring in S (2 epitopes) and N (2 epitopes). These epitopes were not conserved among each other and were not conserved with any epitopes in the CCCoVs (S3 Data).

One region, corresponding to SARS-CoV-2 epitope 807-S-26, showed binding or potential cross-reactivity across all CoVs, and one, corresponding to SARS-CoV-2 epitope 1140-S-25, showed binding or potential cross-reactivity across all β-CoVs (Fig 5). Epitope 807-S-26 includes the CoV S fusion peptide, and 1140-S-25 is immediately adjacent to the heptad repeat region 2, both of which are involved in membrane fusion [42].

### Enzyme-linked immunosorbent assays (ELISAs) confirm peptide microarray findings

**Fig 6:**
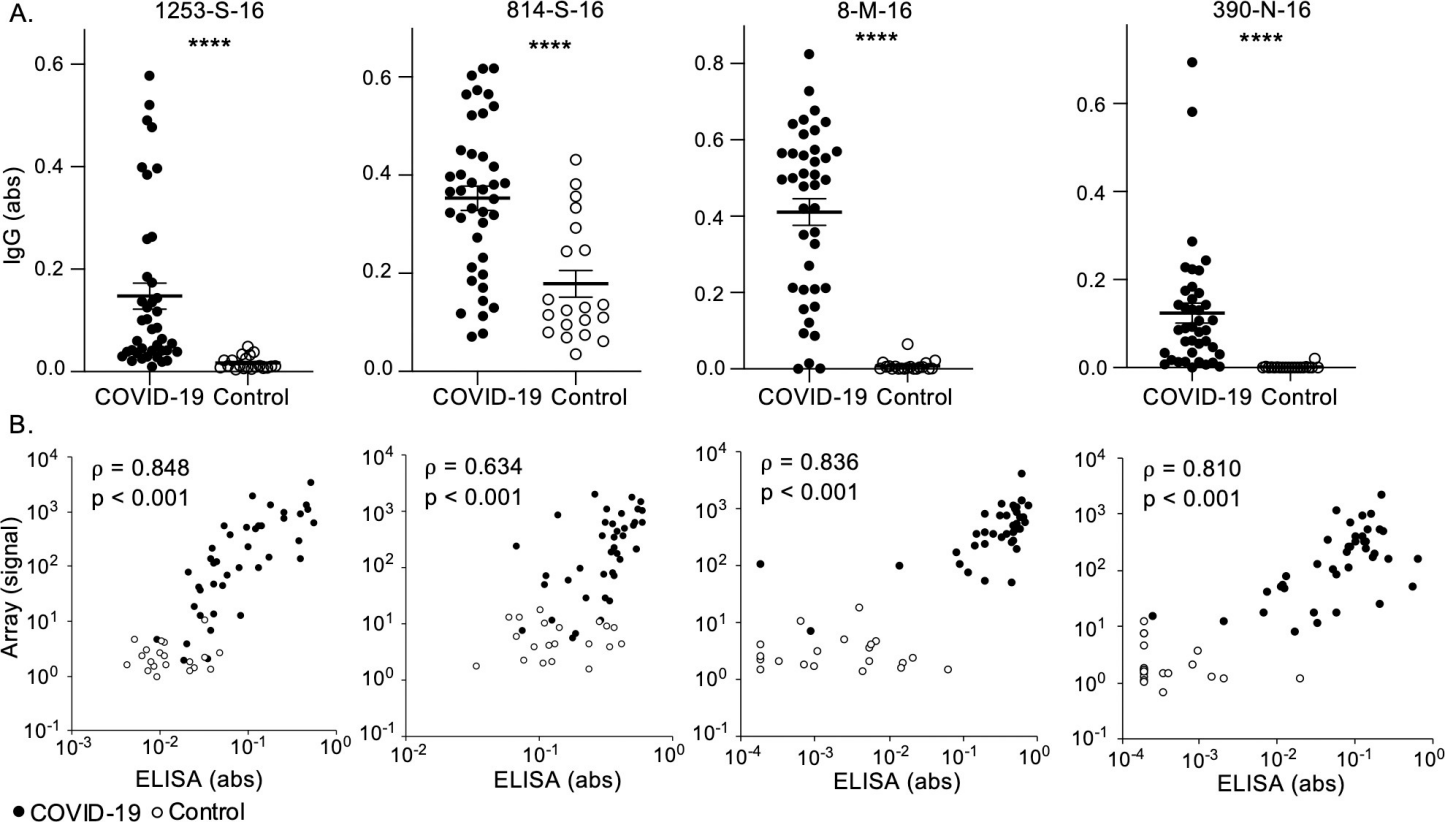
Higher IgG binding to SARS-CoV-2 peptides in COVID-19 convalescent patients compared to controls by ELISA. (A) IgG binding to SARS-CoV-2 peptides in COVID-19 convalescent (*n* = 40) and naïve control (*n =* 20) sera was measured by ELISA. Bars indicate mean abs +/− SEM and ****p < 0.0001 by *t* test. (B) Anti-SARS-CoV-2 peptide IgG detected by ELISA was compared to array findings by Spearman rank-order correlation (Spearman correlation coefficient, ρ) for COVID-19 convalescent (*n =* 40, closed circles) and control (*n =* 20, open circles) sera. The data used in this analysis can be accessed online at: https://github.com/Ong-Research/UW_Adult_Covid-19. abs, absorbance; COVID-19, coronavirus disease 2019; ELISA, enzyme-linked immunosorbent assay; IgG, immunoglobulin G; SARS-CoV-2, severe acute respiratory syndrome coronavirus 2; SEM, standard error of the mean.

Having determined reactivity and apparent cross-reactivity by peptide array, we aimed to independently confirm and validate these findings by ELISA. We selected 4 peptides for ELISA evaluation (1253-S-16, 814-S-16, 8-M-16, and 390-N-16) from those in our top 10 ranked epitopes, considering diversity among the proteins represented, association with neutralizing capacity, and potential cross-reactivity across multiple CoVs, and using the 16-mer in each epitope that most correctly discriminated between patients and controls. All 4 SARS-CoV-2 peptides had higher IgG binding in COVID-19 convalescent sera than in controls (Fig 6). Peptide 8-M-16 showed the greatest discrimination between COVID-19 convalescent and control sera with only 3 COVID-19 convalescent samples having values similar to controls. Both peptides 1253-S-16 and 814-S-16 showed greater binding in controls than either 8-M-16 or 390-N-16, confirming our findings of greater potential cross-reactivity among epitopes found in S.

### Reactivity in some epitopes correlates with disease severity

**Fig 7:**
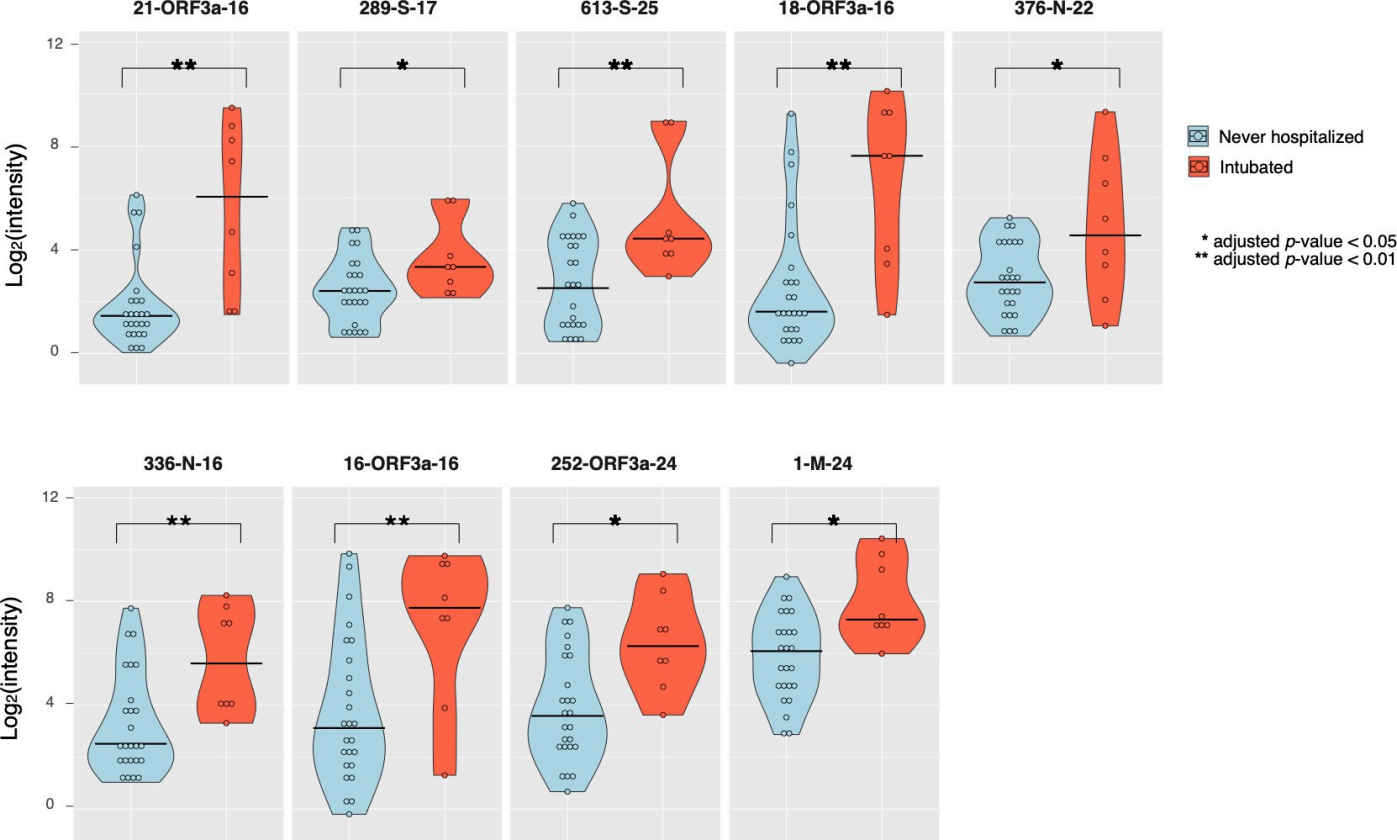
Disease severity correlates with increased antibody binding in specific SARS-CoV-2 epitopes. IgG reactivity against SARS-CoV-2 epitopes identified by peptide microarray in COVID-19 convalescent patients who were never hospitalized versus intubated patients showed statistically significant increases in reactivity in intubated patients for 11 epitopes. The data used in this analysis can be accessed online at: https://github.com/Ong-Research/UW_Adult_Covid-19. COVID-19, coronavirus disease 2019; IgG, immunoglobulin G; SARS-CoV-2, severe acute respiratory syndrome coronavirus 2.

Increased antibody titer and duration have been associated with increased severity of illness due to infection with SARS-CoV-2 [43–47] and other CoVs [48], although data on epitope-level differences by severity is lacking [49]. We compared reactivity in patients within our cohort whose COVID-19 course required intubation and mechanical ventilation (*n =* 8) with reactivity in COVID-19 convalescent patients who never required hospitalization (*n =* 25) using multilinear regression accounting for age, sex, immunocompromising conditions, and Charlson comorbidity index score [50] to determine epitope-level resolution of differences in reactivity. Nine epitopes in S (2 epitopes), M (1 epitope), N (2 epitopes), and ORF3a (4 epitopes) showed statistically significant (*p* < 0.05) increases in reactivity for intubated patients relative to never-hospitalized patients (Fig 7, S4 Table). The S epitopes (289-S-17 and 613-S-25) both occurred in the S1 subunit (aa 14–685), with one (289-S-17) in the N-terminal domain [6] (see Fig 4D), whose function is not well understood but which may play a role in membrane fusion [51]. The M epitope (1-M-24) was the highly reactive epitope in the N-terminus of this protein discussed above. The N epitopes (336-N-16 and 376-N-22) occurred in the C-terminal domain (336-N-16), which is thought to bind nucleic acids, and in the unstructured C-tail (376-N-22) [52]. The ORF3a epitopes clustered near the N-terminus of the protein (16-ORF3a-16, 18-ORF3a-16, and 21-ORF3a-16) with one other epitope nearer the C-terminus (252-ORF3a-24). No epitopes showed statistically significant increases in reactivity for never-hospitalized patients relative to intubated patients (S4 Table).

## Discussion

In our analysis of antibody binding to the full proteome of SARS-CoV-2, the highest magnitude binding of anti-SARS-CoV-2 antibodies from human sera occurred for an epitope in the N-terminus of M protein, with high specificity and sensitivity. Antibodies produced after infection with SARS-CoV-2 reacted with epitopes throughout the proteomes of other human and nonhuman CoVs, recognizing homologous regions across all CoVs. Taken together, these results confirm that humans mount strong, broad antibody responses to SARS-CoV-2 proteins in addition to S and N, and they implicate M epitopes as highly relevant to diagnostic and potentially to vaccine design.

M proteins are the most abundant proteins in CoV virions [17]. The N-terminus of M is known in other CoVs to be a small, glycosylated ectodomain that protrudes outside the virion and interacts with S, N, and E [17], while the rest of M resides within the viral particle. Full-length SARS-CoV M has been shown to induce protective antibodies [20,53], and patterns of antibodies binding to SARS-CoV M are similar to those we found in SARS-CoV-2 [35]. SARS-CoV anti-M antibodies can synergize with anti-S and anti-N antibodies for improved neutralization [20,53], and M has been used in protective SARS-CoV and MERS-CoV vaccines [8]. However, the mechanism of protection of anti-M antibodies remains unknown, and this protein remains largely understudied and underutilized as an antigen. Other groups have not previously identified the high magnitude binding we observed for M, though that may be due to other studies’ use of samples collected earlier in the course of infection or different techniques, populations, or computational algorithms [54,55]. Notably, some of the highest binding we observed in the S protein occurred at the base of the extracellular portion of the protein, which would be the site of the putative interaction between SARS-CoV-2 S and M. The ACE2 binding site and the RBD in general are not as reactive, by these methods, as expected, suggesting that other, less-investigated epitopes may be playing a larger role in immunity to SARS-CoV-2 than is currently appreciated, which is further bolstered by the correlation of some of this binding with neutralizing titers. Our results, in concert with prior knowledge of anti-SARS-CoV antibodies, strongly suggest that epitopes in M, particularly the 1-M-24 epitope as well as other novel epitopes we identified, should be investigated further as potential targets in SARS-CoV-2 diagnostics, vaccines, and therapeutics.

Among the accessory proteins against which we detected antibodies, ORF8 has been the best studied. The *ORF8* gene is part of a hypervariable region, having undergone multiple substitutions and deletions and being recognized as a recombination hotspot [56]. ORF8 protein is considered to have immunomodulatory activity and has been shown to potently down-regulate major histocompatibility complex class I expression in several cell lines [57] and to antagonize interferon signaling [58,59]. A deletion in ORF8 appears to be associated with a milder clinical COVID-19 course [60], and ORF8 has been shown to be secreted [61], indicating that the epitopes we defined here may merit further investigation for development of potential vaccines and therapeutics. Less is known about the other SARS-CoV-2 accessory proteins in which we found epitopes, ORF3a and ORF6, although some studies have implicated them in immunomodulatory functions [59,62–64]. Further investigations will be needed to determine the function of these proteins, which will provide greater insight into how the epitopes we found may be useful in the development of countermeasures against COVID-19.

Interestingly, we found antibodies bind or bind adjacent to a number of the mutations in some of the “variants of concern” (VOCs) of SARS-CoV-2, which have recently emerged [65–67], so named because they appear to potentially be more transmissible than previous known variants or to escape antibody binding [68]. The epitopes we defined contained or were immediately adjacent to the locations of the majority of the variant-defining mutation sites in the structural and accessory proteins of the B.1.1.7 and B.1.351 variants and to one-quarter of the structural or accessory protein mutations sites of the P.1, B.1.427, and B.1.429 variants (see S5 Table). These results suggest that antigen escape may be driving the rise and dominance of variants. Recent works have demonstrated this phenomenon with mutations in S [69,70] but have not investigated this possibility for other proteins. Our findings suggest that antibodies to non-S proteins may be important to this process, as well. Given that the VOCs were identified after we performed our experiments, we did not include mutations characteristic of the VOCs in our array design for this current work. Future studies should include representation of the VOCs in order to discern any differences in antibody binding.

We also found that antibodies produced in response to SARS-CoV-2 infection appear to bind peptides representing homologous epitopes throughout the proteomes of other human and nonhuman CoVs. Hundreds of CoVs have been discovered in bats and other species [27,39–41,71,72], making future spillovers inevitable. The potential broad cross-reactivity we observed in some homologous peptide sequences may help guide the development of pan-CoV vaccines [15], especially given that antibodies binding to 807-S-26 and 1140-S-25, which showed potential cross-reactivity across all CoVs and all β-CoVs, respectively, are known to be potently neutralizing [32,33]. A caveat is that our methods cannot discern whether the increased IgG binding to CCCoVs in COVID-19 convalescent sera is due to newly developed cross-reactive antibodies or due to the stimulation of a memory response against the original CCCoV antigens. However, cross-reactivity of anti-SARS-CoV-2 antibodies with SARS-CoV or MERS-CoV is likely real, since our population was very unlikely to have been exposed to those viruses. A more stringent assessment of cross-reactivity as well as functional investigations into these cross-reactive antibodies will be vital in determining their capacity for cross-protection. Further, our methods efficiently detect antibody binding to linear epitopes [73], but their sensitivity for detecting parts of conformational epitopes, which are considered highly important in the immune response to SARS-CoV-2 and which are believed to be the type of epitope found within the RBD [23,25,74–76], is unknown. Additional analyses will be required to determine whether epitopes newly identified here induce neutralizing or otherwise protective antibodies. It is interesting to note that SARS-CoV-2 infection resulted in some antibodies that bound epitopes in other coronaviruses without binding the homologous part of SARS-CoV-2. These epitopes were not conserved with each other (S3 Data), and most of this binding occurred in nonstructural proteins in ORF1ab, which may indicate that this was nonspecific binding resulting from a generalized immune activation.

Finally, we demonstrated that more severely ill patients have significantly greater reactivity to certain epitopes in S, M, N, and ORF3a. The 9 epitopes with significantly higher magnitude reactivity in intubated patients may play a role in the overaggressive immune response known to characterize severe COVID-19 [7,77], suggesting that they may be targets for treatment in or prevention of severe disease. Our data collection included date of first positive test (S1 Table) but not of symptom onset, but future studies that include these data could investigate potential correlations between symptoms and antibody kinetics. Alternatively, the antibody response in general may be higher in very sick patients, expanding the repertoire of antibody reactivity. Future studies should investigate whether these differences can be detected early in the disease course to determine their potential utility as predictive markers of disease severity. Future studies may also investigate these epitopes’ potential as targets for medical countermeasures [51], although consideration should be given to the small sample size of our investigation.

Many questions remain regarding the biology and immunology related to SARS-CoV-2. Our extensive profiling of epitope-level resolution antibody reactivity in COVID-19 convalescent patients, confirmed by independent assays, provides new epitopes that could serve as important targets in the development of improved diagnostics, vaccines, and therapeutics against SARS-CoV-2 and dangerous human CoVs that may emerge in the future.

## Methods

### Peptide microarray design and synthesis

Viral protein sequences were selected and submitted to Nimble Therapeutics (Madison, Wisconsin, USA) for development into a peptide microarray [73]. Sequences represented include proteomes of all 7 coronaviruses known to infect humans, proteomes of closely related coronaviruses found in bats and pangolins, and spike proteins from other coronaviruses (accession numbers and replicates per peptide shown in S5 Data). A number of proteins were included as controls, including poliovirus, 7 strains of human rhinovirus, and human cytomegalovirus 65kDa phosphoprotein. We chose these controls given that we expect most human adults will have antibody reactivity to at least one of these proteins and proteomes. Accession numbers used to represent each viral protein are listed in the Supporting information (accession numbers and replicates per peptide shown in S5 Data). All proteins were tiled as 16 amino acid peptides overlapping by 15 amino acids. All unique peptides were tiled in a lawn of thousands of copies, with each unique peptide represented in at least 3 and up to 5 replicates (S5 Data). The peptide sequences were synthesized in situ with a Nimble Therapeutics Maskless Array Synthesizer (MAS) by light-directed solid-phase peptide synthesis using an amino-functionalized support (Geiner Bio-One) coupled with a 6-aminohexanoic acid linker and amino acid derivatives carrying a photosensitive 2-(2-nitrophenyl) propyloxycarbonyl (NPPOC) protection group (Orgentis Chemicals). Unique peptides were synthesized in random positions on the array to minimize impact of positional bias. Each array consists of 12 subarrays, where each subarray can process 1 sample, and each subarray contains up to 389,000 unique peptide sequences.

### Human patients and controls

The study was conducted in accordance with the Declaration of Helsinki and approved by the Institutional Review Board of the University of Wisconsin-Madison. Clinical data and sera from patients infected with SARS-CoV-2 were obtained from the University of Wisconsin (UW) COVID-19 Convalescent Biobank and from controls (sera collected prior to 2019) from the UW Rheumatology Biobank [78]. All patients and controls were 18 years of age or older at the time of recruitment and provided informed consent. COVID-19 convalescent patients had a positive SARS-COV-2 PCR test at UW Health with sera collected 5 to 6 weeks after self-reported COVID-19 symptom resolution except blood was collected for 1 patient after 9 weeks. Age, sex, medications, and medical problems were abstracted from UW Health’s electronic medical record (EMR). Race and ethnicity were self-reported. Hospitalization and intubation for COVID-19 and smoking status at the time of blood collection (controls) or COVID-19 were obtained by EMR abstraction and self-report and were in complete agreement. Two-thirds of COVID-19 convalescent patients and all controls had a primary care appointment at UW Health within 2 years of the blood draw as an indicator of the completeness of the medical information. Patients and controls were considered to have an immunocompromising condition if they met any of the following criteria: immunosuppressing medications, systemic inflammatory or autoimmune disease, cancer not in remission, uncontrolled diabetes (secondary manifestations or hemoglobin A1c >7.0%), or congenital or acquired immunodeficiency. Controls and COVID-19 patients were similar in regard to demographics and health (S5 Data), and patients who were not hospitalized, were hospitalized, or were hospitalized and intubated also were compared (S5 Data). No patients or controls were current smokers.

### Peptide array sample binding

Samples were diluted 1:100 in binding buffer (0.01 M Tris-Cl (pH 7.4), 1% alkali-soluble casein, 0.05% Tween-20) and bound to arrays overnight at 4°C. After sample binding, the arrays were washed 3× in wash buffer (1× TBS, 0.05% Tween-20), 10 minutes per wash. Primary sample binding was detected via Alexa Fluor 647-conjugated goat anti-human IgG secondary antibody (Jackson ImmunoResearch, West Grove, Pennsylvania, United States of America). The secondary antibody was diluted 1:10,000 (final concentration 0.1 ng/μl) in secondary binding buffer (1× TBS, 1% alkali-soluble casein, 0.05% Tween-20). Arrays were incubated with secondary antibody for 3 hours at room temperature, then washed 3× in wash buffer (10 minutes per wash), washed for 30 seconds in reagent-grade water, and then dried by spinning in a microcentrifuge equipped with an array holder. The fluorescent signal of the secondary antibody was detected by scanning at 635 nm at 2 μm resolution using an Innopsys 910AL microarray scanner. Scanned array images were analyzed with proprietary Nimble Therapeutics software to extract fluorescence intensity values for each peptide.

### Peptide microarray findings validation

We included sequences on the array of viruses that we expected all adult humans to be likely to have been exposed to as positive controls: 1 poliovirus strain (measuring vaccine exposure) and 7 rhinovirus strains. Any patient or control whose sera did not react to at least 1 positive control would be considered a failed run and removed from the analysis. All patients and controls in this analysis reacted to epitopes in at least 1 control strain (Fig 1, S1 Data).

### Peptide microarray data analysis

The raw fluorescence signal intensity values were log_2_ transformed. Clusters of fluorescence intensity of statistically unlikely magnitude, indicating array defects, were identified and removed. Local and large area spatial corrections were applied, and the median transformed intensity of the peptide replicates was determined. The resulting median data was cross-normalized using quantile normalization.

### Neutralization assay

Virus neutralization assays were performed with SARS-CoV-2/UW-001/Human/2020/Wisconsin on Vero E6/TMPRSS2 [79]. Virus (approximately 100 plaque-forming units) was incubated with the same volume of 2-fold dilutions of heat-inactivated serum for 30 minutes at 37°C. The antibody/virus mixture was added to confluent Vero E6/TMPRSS2 cells that were plated at 30,000 cells per well the day prior in 96-well plates. The cells were incubated for 3 days at 37°C and then fixed and stained with 20% methanol and crystal violet solution. Virus neutralization titers were determined as the reciprocal of the highest serum dilution that completely prevented cytopathic effects.

### Protein structures

The SARS-CoV-2 S-chimera.pdb used to make S protein structures is a chimeric structure built by Robert Kirchdoerfer using 6VYB.pdb, 5X4S.pdb, and 6LZG coordinates and filling in internal unresolved residues from known (presumably) analogous sites determined for SARS-CoV S from 6CRV.pdb. Additional unmodeled regions were generated using Modeller [80]. C-proximal HR2 regions were modeled as single helices (Phe1148-Leu1211) in Coot [81].

The data2bfactor Python script written by Robert L. Campbell, Thomas Holder, and Suguru Asai (downloaded from http://pldserver1.biochem.queensu.ca/~rlc/work/pymol/) was used to substitute peptide array data onto this structure in place of the B factor in PyMol (The PyMOL Molecular Graphics System, Version 2.0 Schrödinger, LLC) using a dark blue (low) to red (high) color scale. Data used for these visualizations were the average reactivity in the 40 COVID-19 convalescent patients, the average reactivity in the 20 naïve controls, and the difference between averages for the patients and for the controls.

### Enzyme-linked immunosorbent assays (ELISAs)

Costar 96-well high-binding plates (Corning, Corning, USA) were incubated at 4°C overnight with 5 μg/ml streptavidin (Thermo Fisher Scientific, Waltham, USA) in PBS (Corning). Plates were washed twice with PBS and incubated at room temperature for 1 hour with 0.5 mM of the following peptides (Biomatik, Kitchener, Canada) in PBS: 814-S-16 (KRSFIEDLLFNKVTLA-K-biotin), 1253-S-16 (CCKFDEDDSEPVLKGV-K-biotin), 390-N-16 (QTVTLLPAADLDDFSK-K-biotin), and 8-M-16 (ITVEELKKLLEQWNLV-K-biotin). Plates were washed thrice with wash buffer (0.2% Tween-20 in PBS), then incubated for 1 hour in blocking solution (5% nonfat dry milk in wash buffer) at room temperature, incubated overnight at 4°C with sera at 1:200 in blocking solution, washed 4 times with wash buffer, incubated for 1 hour at room temperature with mouse anti-human IgG conjugated to horse radish peroxidase (Southern Biotech, Birmingham, USA) diluted 1:5,000 in blocking solution, washed 4 times with wash buffer, and incubated with tetramethyl benzidine substrate solution (Thermo Fisher Scientific) for 5 minutes followed by 0.18 M sulfuric acid. Absorbance was read on a FilterMax F3 Multi-mode Microplate reader (Molecular Devices, San Jose, USA) at 450 and 562 nm. Background signal from 562 nm absorbance and wells with no peptide and no serum were subtracted. Plates were normalized using a pooled serum sample on every plate. Absorbance values of 0 were plotted as 0.0002 to allow a log scale for graphs. Samples were run in duplicate.

### Statistical analysis

Statistical analyses were performed in R (v 4.0.2) using in-house scripts. For each peptide, a *p*-value from a two-sided *t* test with unequal variance between sets of patient and control responses were calculated and adjusted using the Benjamini–Hochberg (BH) algorithm. To determine whether the peptide was in an epitope (in SARS-CoV-2 proteins) or cross-reactive for anti-SARS-CoV-2 antibodies (in non-SARS-CoV-2 proteins), we used an adjusted *p*-value cutoff of <0.1 (based on multiple hypothesis testing correction for all 119,487 unique sequences on the array) and a fold-change of greater than or equal to 2 and grouped consecutive peptides as a represented epitope. Linear discriminant analysis leave-one-out cross validation was used to determine specificity and sensitivity on each peptide and from each epitope using the average signal of the component peptides. Pearson correlation for reactivity with neutralizing titer was calculated using each patient’s or control’s epitope signal and the log_2_ signal of the respective neutralization value.

To identify cross-reactive epitopes, we used each SARS-CoV-2 epitope sequence as a query, searched the database of proteins from the sequences in the peptide array using blastp (-word-size 2, num-targets 4,000) to find homologous sequences in the bat, pangolin, and other human CoV strains, then determined whether the average log_2_-normalized intensity for these sequences in patients was at least 2-fold greater than in controls with *t* test statistics yielding adjusted *p*-values <0.1. Each blast hit was then mapped back to the corresponding probe ranges.

For correlations of reactivity with clinical severity, for each patient, the epitope signal was determined by averaging the normalized signal from the epitopes corresponding probes. Each epitope average signal response was fit using a multilinear regression model accounting for age, sex (Female, Male), immunocompromised status (Yes, No), and Charlson comorbidity index score [50] as additive. Contrasts between nonhospitalized and intubated patients were performed for each epitope with the fit models and *p*-values and log_2_ fold-change were determined.

The clinical and demographic characteristics of convalescent patients were compared to those of the controls using χ2 tests for categorical variables and Wilcoxon rank-sum tests for nonnormally distributed continuous measures.

Heatmaps were created using the gridtext [82] and complexheatmap [83] packages in R. Alignments for heatmaps were created using MUSCLE [84].

## Supporting information

S1 Fig Anti-SARS-CoV-2 antibody binding patterns do not vary with neutralizing titer. Sera from 40 COVID-19 convalescent patients were assayed for IgG binding to the full SARS-CoV-2 proteome on a peptide microarray. B cell epitopes were defined as peptides in which patients’ average log_2_-normalized intensity (black lines in line plots) is 2-fold greater than controls’ (gray lines in line plots) and *t* test statistics yield adjusted *p*-values <0.1; epitopes are identified by orange shading in the line plots. Data are grouped by their neutralizing titer. COVID-19, coronavirus disease 2019; IgG, immunoglobulin G; SARS-CoV-2, severe acute respiratory syndrome coronavirus 2.
(XLSX)

S2 Fig Alignment of epitopes in human and animal CoVs for which antibodies in sera from 40 COVID-19 convalescent patients showed apparent cross-reactive binding. Alignments were performed in Geneious Prime 2020.1.2 (Auckland, New Zealand). CoV, coronavirus; COVID-19, coronavirus disease 2019.
(PDF)

S1 Table Metadata for the 40 COVID-19 convalescent patients and 20 naïve controls: Patient or control status, age at blood draw, legal sex, Charlson comorbidity index, immunocompromised status, whether the COVID-19 convalescent patients required hospitalization or intubation for their COVID-19 course, race, and ethnicity. Neutralizing titers and the dates of the first positive COVID-19 test are also included. COVID-19, coronavirus disease 2019.
(XLSX)

S2 Table Specificity and sensitivity for past SARS-CoV-2 infection in 40 COVID-19 convalescent patients compared to 20 naïve controls of individual 16-mer peptides comprising epitopes throughout the full SARS-CoV-2 proteome. COVID-19, coronavirus disease 2019; SARS-CoV-2, severe acute respiratory syndrome coronavirus 2.
(XLSX)

S3 Table Epitopes paired with the 1-M-24 epitope obtained AUC-ROC of 1.0 for SARS-CoV-2 infection in 40 COVID-19 convalescent patients and 20 naïve controls using leave-one-out cross validation with linear discriminant analysis. AUC-ROC, area under the receiver operating characteristic curve; COVID-19, coronavirus disease 2019; SARS-CoV-2, severe acute respiratory syndrome coronavirus 2.
(XLSX)

S4 Table Comparison of antibody binding in SARS-CoV-2 B cell epitopes in 8 intubated COVID-19 convalescent patients compared to 25 symptomatic but never hospitalized COVID-19 convalescent patients compared by multilinear regression accounting for age, sex, immunocompromising conditions, and Charlson comorbidity index score. COVID-19, coronavirus disease 2019; SARS-CoV-2, severe acute respiratory syndrome coronavirus 2.
(XLSX)

S5 Table B cell epitopes detected by sera from 40 individuals having a first positive SARS-CoV-2 test in March through May 2020 overlap with protein areas containing in SARS-CoV-2 variants of concern. SARS-CoV-2, severe acute respiratory syndrome coronavirus 2.
(XLSX)

S1 Data Percentages and individual reactivity of the 40 COVID-19 convalescent patients and 20 naïve controls reacted to known epitopes in at least 1 control virus (rhinovirus and poliovirus strains). COVID-19, coronavirus disease 2019.
(XLSX)

S2 Data Percentages and individual data for the 40 COVID-19 convalescent patients and 20 naïve controls showing log_2_-normalized fluorescence intensity at least 3.00 standard deviations above the mean for the array for 9 species of CoVs. CoV, coronavirus; COVID-19, coronavirus disease 2019.
(XLSX)

S3 Data Cross-reactive binding of antibodies against other CoVs in 40 COVID-19 convalescent patients compared to 20 naïve controls. CoV, coronavirus; COVID-19, coronavirus disease 2019.
(XLSX)

S4 Data Cross-reactive binding of antibodies in 40 COVID-19 convalescent patients compared to 20 naïve controls in protein motifs in other CoVs aligned to SARS-CoV-2. CoV, coronavirus; COVID-19, coronavirus disease 2019; SARS-CoV-2, severe acute respiratory syndrome coronavirus 2.
(XLSX)

S5 Data Supporting information tables (3 tables) show data relevant to the Methods for this study. Supporting information Table A contains the proteins represented on the array, including the GenBank accession numbers and the number of replicates of reach peptide in those proteins. Supporting information Table B contains the characteristics of the 40 COVID-19 convalescent patients and the 20 naïve controls whose sera were used in this study. Supporting information Table C contains the characteristics of the 40 COVID-19 convalescent patients according to hospitalization status. COVID-19, coronavirus disease 2019.
(DOCX)

## Abbreviations

ACE2: angiotensin converting enzyme 2
β-CoV: betacoronavirus
BH: Benjamini–Hochberg
CCCoVs: “common cold” CoVs
CoV: coronavirus
COVID-19: coronavirus disease 2019
E: envelope
ELISA: enzyme-linked immunosorbent assay
EMR: electronic medical record
IgG: immunoglobulin G
M: membrane
MAS: Maskless Array Synthesizer
MERS-CoV: Middle Eastern respiratory syndrome coronavirus
N: nucleocapsid
NPPOC: 2-(2-nitrophenyl) propyloxycarbonyl
S: spike
PBS: phosphate-buffered saline
RBD: receptor-binding domain
SARS-CoV: severe acute respiratory syndrome coronavirus
SARS-CoV-2: severe acute respiratory syndrome coronavirus 2
TBS: tris-buffered saline
UW: University of Wisconsin
VOC: variant of concern

## References

[1] Deeks JJ, Dinnes J, Takwoingi Y, Davenport C, Spijker R, Taylor-Phillips S, et al. Antibody tests for identification of current and past infection with SARS-CoV-2. Cochrane Database Syst Rev. 2020;6:CD013652. doi: 10.1002/14651858.CD013652 32584464 PMC7387103

[2] Liu W, Liu L, Kou G, Zheng Y, Ding Y, Ni W, et al. Evaluation of Nucleocapsid and Spike Protein-Based Enzyme-Linked Immunosorbent Assays for Detecting Antibodies against SARS-CoV-2. J Clin Microbiol. 2020;58. doi: 10.1128/JCM.00461-20 32229605 PMC7269413

[3] Tré-Hardy M, Wilmet A, Beukinga I, Favresse J, Dogne JM, Douxfils J, et al. Analytical and clinical validation of an ELISA for specific SARS-CoV-2 IgG, IgA, and IgM antibodies. J Med Virol. 2020. 10.1002/jmv.26303 PMC7405491 32667733

[4] Lisboa Bastos M, Tavaziva G, Abidi SK, Campbell JR, Haraoui LP, Johnston JC, et al. Diagnostic accuracy of serological tests for covid-19: systematic review and meta-analysis. BMJ. 2020;m2516:370. doi: 10.1136/bmj.m2516 32611558 PMC7327913

[5] Ayouba A, Thaurignac G, Morquin D, Tuaillon E, Raulino R, Nkuba A, et al. Multiplex detection and dynamics of IgG antibodies to SARS-CoV2 and the highly pathogenic human coronaviruses SARS-CoV and MERS-CoV. J Clin Virol. 2020;129:104521. doi: 10.1016/j.jcv.2020.104521 32623350 PMC7308014

[6] Huang Y, Yang C, Xu XF, Xu W, Liu SW. Structural and functional properties of SARS-CoV-2 spike protein: potential antivirus drug development for COVID-19. Acta Pharmacol Sin. 2020;41:1141–9. doi: 10.1038/s41401-020-0485-4 32747721 PMC7396720

[7] Chen W. Promise and challenges in the development of COVID-19 vaccines. Hum Vaccin Immunother. 2020 1–5. doi: 10.1080/21645515.2020.1787067 32703069 PMC7733917

[8] Ong E, Wong MU, Huffman A, He Y. COVID-19 Coronavirus Vaccine Design Using Reverse Vaccinology and Machine Learning. Front Immunol. 2020;11:1581. doi: 10.3389/fimmu.2020.01581 32719684 PMC7350702

[9] Sui J, Li W, Murakami A, Tamin A, Matthews LJ, Wong SK, et al. Potent neutralization of severe acute respiratory syndrome (SARS) coronavirus by a human mAb to S1 protein that blocks receptor association. Proc Natl Acad Sci U S A. 2004;101:2536–41. doi: 10.1073/pnas.0307140101 14983044 PMC356985

[10] ter Meulen J, Bakker AB, van den Brink EN, Weverling GJ, Martina BE, Haagmans BL, et al. Human monoclonal antibody as prophylaxis for SARS coronavirus infection in ferrets. Lancet. 2004;363:2139–41. doi: 10.1016/S0140-6736(04)16506-9 15220038 PMC7112500

[11] Rockx B, Corti D, Donaldson E, Sheahan T, Stadler K, Lanzavecchia A, et al. Structural basis for potent cross-neutralizing human monoclonal antibody protection against lethal human and zoonotic severe acute respiratory syndrome coronavirus challenge. J Virol. 2008;82:3220–35. doi: 10.1128/JVI.02377-07 18199635 PMC2268459

[12] Martin JE, Louder MK, Holman LA, Gordon IJ, Enama ME, Larkin BD, et al. A SARS DNA vaccine induces neutralizing antibody and cellular immune responses in healthy adults in a Phase I clinical trial. Vaccine. 2008;26:6338–43. doi: 10.1016/j.vaccine.2008.09.026 18824060 PMC2612543

[13] Jiang L, Wang N, Zuo T, Shi X, Poon KM, Wu Y, et al. Potent neutralization of MERS-CoV by human neutralizing monoclonal antibodies to the viral spike glycoprotein. Sci Transl Med. 2014;6:234ra59. doi: 10.1126/scitranslmed.3008140 24778414

[14] Chen Z, Bao L, Chen C, Zou T, Xue Y, Li F, et al. Human Neutralizing Monoclonal Antibody Inhibition of Middle East Respiratory Syndrome Coronavirus Replication in the Common Marmoset. J Infect Dis. 2017;215:1807–15. doi: 10.1093/infdis/jix209 28472421 PMC7107363

[15] Burton DR, Walker LM. Rational Vaccine Design in the Time of COVID-19. Cell Host Microbe. 2020;27:695–8. doi: 10.1016/j.chom.2020.04.022 32407707 PMC7219357

[16] Zhou Y, Jiang S, Du L. Prospects for a MERS-CoV spike vaccine. Expert Rev Vaccines. 2018;17:677–86. doi: 10.1080/14760584.2018.1506702 30058403 PMC6355461

[17] Maier HJ, Bickerton E, Britton P. Coronaviruses: methods and protocols. New York: Humana Press; Springer; 2015:xi, 285 pages.

[18] Ravichandran S, Coyle EM, Klenow L, Tang J, Grubbs G, Liu S, et al. Antibody signature induced by SARS-CoV-2 spike protein immunogens in rabbits. Sci Transl Med. 2020;12. doi: 10.1126/scitranslmed.abc3539 32513867 PMC7286538

[19] Knoll MD, Wonodi C. Oxford-AstraZeneca COVID-19 vaccine efficacy. Lancet. 2020. doi: 10.1016/S0140-6736(20)32623-4 33306990 PMC7832220

[20] Pang H, Liu Y, Han X, Xu Y, Jiang F, Wu D, et al. Protective humoral responses to severe acute respiratory syndrome-associated coronavirus: implications for the design of an effective protein-based vaccine. J Gen Virol. 2004;85:3109–13. doi: 10.1099/vir.0.80111-0 15448374

[21] Chow SC, Ho CY, Tam TT, Wu C, Cheung T, Chan PK, et al. Specific epitopes of the structural and hypothetical proteins elicit variable humoral responses in SARS patients. J Clin Pathol. 2006;59:468–76. doi: 10.1136/jcp.2005.029868 16461566 PMC1860290

[22] He Y, Zhou Y, Siddiqui P, Niu J, Jiang S. Identification of immunodominant epitopes on the membrane protein of the severe acute respiratory syndrome-associated coronavirus. J Clin Microbiol. 2005;43:3718–26. doi: 10.1128/JCM.43.8.3718-3726.2005 16081901 PMC1234014

[23] Tian X, Li C, Huang A, Xia S, Lu S, Shi Z, et al. Potent binding of 2019 novel coronavirus spike protein by a SARS coronavirus-specific human monoclonal antibody. Emerg Microbes Infect. 2020;9(1):382–5. doi: 10.1080/22221751.2020.1729069 32065055 PMC7048180

[24] Lv H, Wu NC, Tsang OT, Yuan M, Perera R, Leung WS, et al. Cross-reactive Antibody Response between SARS-CoV-2 and SARS-CoV Infections. Cell Rep. 2020;31:107725. 10.1016/j.celrep.2020.107725 PMC7231734 32426212

[25] Pinto D, Park YJ, Beltramello M, Walls AC, Tortorici MA, Bianchi S, et al. Cross-neutralization of SARS-CoV-2 by a human monoclonal SARS-CoV antibody. Nature. 2020;583:290–5. doi: 10.1038/s41586-020-2349-y 32422645

[26] Nickbakhsh S, Ho A, Marques DFP, McMenamin J, Gunson RN, Murcia PR. Epidemiology of Seasonal Coronaviruses: Establishing the Context for the Emergence of Coronavirus Disease 2019. J Infect Dis. 2020;222:17–25. doi: 10.1093/infdis/jiaa185 32296837 PMC7184404

[27] Morens DM, Fauci AS. Emerging Pandemic Diseases: How We Got to COVID-19. Cell. 2020;182:1077–92. doi: 10.1016/j.cell.2020.08.021 32846157 PMC7428724

[28] Gorse GJ, Patel GB, Vitale JN, O’Connor TZ. Prevalence of antibodies to four human coronaviruses is lower in nasal secretions than in serum. Clin Vaccine Immunol. 2010;17:1875–80. doi: 10.1128/CVI.00278-10 20943876 PMC3008199

[29] Premkumar L, Segovia-Chumbez B, Jadi R, Martinez DR, Raut R, Markmann A, et al. The receptor binding domain of the viral spike protein is an immunodominant and highly specific target of antibodies in SARS-CoV-2 patients. Sci Immunol. 2020:5. doi: 10.1126/sciimmunol.abc8413 32527802 PMC7292505

[30] Valentini D, Rao M, Rane L, Rahman S, Axelsson-Robertson R, Heuchel R, et al. Peptide microarray-based characterization of antibody responses to host proteins after bacille Calmette-Guérin vaccination. Int J Infect Dis. 2017;56:140–54. doi: 10.1016/j.ijid.2017.01.027 28161459

[31] Amjadi MF, O’Connell SE, Armbrust T, Mergaert AM, Narpala SR, Halfmann PJ, et al. Specific COVID-19 Symptoms Correlate with High Antibody Levels against SARS-CoV-2. ImmnoHorizons. 2020. doi: 10.4049/immunohorizons.2100022 PMC8452279 34398806

[32] Poh CM, Carissimo G, Wang B, Amrun SN, Lee CY, Chee RS, et al. Two linear epitopes on the SARS-CoV-2 spike protein that elicit neutralising antibodies in COVID-19 patients. Nat Commun. 2020;11:2806. doi: 10.1038/s41467-020-16638-2 32483236 PMC7264175

[33] Zhang BZ, Hu YF, Chen LL, Yau T, Tong YG, Hu JC, et al. Mining of epitopes on spike protein of SARS-CoV-2 from COVID-19 patients. Cell Res. 2020;30(8):702–4. doi: 10.1038/s41422-020-0366-x 32612199 PMC7327194

[34] Li Y, Lai DY, Zhang HN, Jiang HW, Tian X, Ma ML, et al. Linear epitopes of SARS-CoV-2 spike protein elicit neutralizing antibodies in COVID-19 patients. Cell Mol Immunol. 2020;17:1095–7. doi: 10.1038/s41423-020-00523-5 32895485 PMC7475724

[35] Grifoni A, Sidney J, Zhang Y, Scheuermann RH, Peters B, Sette, A. Sequence Homology and Bioinformatic Approach Can Predict Candidate Targets for Immune Responses to SARS-CoV-2. Cell Host Microbe. 2020;27:671–680.e2. doi: 10.1016/j.chom.2020.03.002 32183941 PMC7142693

[36] Ahmed SF, Quadeer AA, McKay MR. Preliminary Identification of Potential Vaccine Targets for the COVID-19 Coronavirus (SARS-CoV-2) Based on SARS-CoV Immunological Studies. Viruses. 2020;12. doi: 10.3390/v12030254 32106567 PMC7150947

[37] Crooke SN, Ovsyannikova IG, Kennedy RB, Poland GA. Immunoinformatic identification of B cell and T cell epitopes in the SARS-CoV-2 proteome. Sci Rep. 2020;10:14179. doi: 10.1038/s41598-020-70864-8 32843695 PMC7447814

[38] Yi C, Sun X, Ye J, Ding L, Liu M, Yang Z, et al. Key residues of the receptor binding motif in the spike protein of SARS-CoV-2 that interact with ACE2 and neutralizing antibodies. Cell Mol Immunol. 2020;17:621–30. doi: 10.1038/s41423-020-0458-z 32415260 PMC7227451

[39] Zhou P, Yang XL, Wang XG, Hu B, Zhang L, Zhang W, et al. A pneumonia outbreak associated with a new coronavirus of probable bat origin. Nature. 2020;579:270–3. doi: 10.1038/s41586-020-2012-7 32015507 PMC7095418

[40] Ge XY, Wang N, Zhang W, Hu B, Li B, Zhang YZ, et al. Coexistence of multiple coronaviruses in several bat colonies in an abandoned mineshaft. Virol Sin. 2016;31:31–40. doi: 10.1007/s12250-016-3713-9 26920708 PMC7090819

[41] Xiao K, Zhai J, Feng Y, Zhou N, Zhang X, Zou JJ, et al. Isolation of SARS-CoV-2-related coronavirus from Malayan pangolins. Nature. 2020;583:286–9. doi: 10.1038/s41586-020-2313-x 32380510

[42] Tang T, Bidon M, Jaimes JA, Whittaker GR, Daniel S. Coronavirus membrane fusion mechanism offers a potential target for antiviral development. Antiviral Res. 2020;178:104792. doi: 10.1016/j.antiviral.2020.104792 32272173 PMC7194977

[43] Behrens GMN, Cossmann A, Stankov MV, Witte T, Ernst D, Happle C, et al. Perceived versus proven SARS-CoV-2-specific immune responses in health-care professionals. Infection. 2020;48:631–4. doi: 10.1007/s15010-020-01461-0 32524515 PMC7286418

[44] Caturegli G, Materi J, Howard BM, Caturegli P. Clinical Validity of Serum Antibodies to SARS-CoV-2: A Case-Control Study. Ann Intern Med. 2020. doi: 10.7326/M20-2889 32628534 PMC7370852

[45] Long QX, Tang XJ, Shi QL, Li Q, Deng HJ, Yuan J, et al. Clinical and immunological assessment of asymptomatic SARS-CoV-2 infections. Nat Med. 2020;26:1200–4. doi: 10.1038/s41591-020-0965-6 32555424

[46] Ko JH, Joo EJ, Park SJ, Baek JY, Kim WD, Jee J, et al. Neutralizing Antibody Production in Asymptomatic and Mild COVID-19 Patients, in Comparison with Pneumonic COVID-19 Patients. J Clin Med. 2020;9. doi: 10.3390/jcm9072268 32708872 PMC7408950

[47] Kirkcaldy RD, King BA, Brooks JT. COVID-19 and Postinfection Immunity: Limited Evidence, Many Remaining Questions. JAMA. 2020;323:2245–6. doi: 10.1001/jama.2020.7869 32391855 PMC8596300

[48] Huang AT, Garcia-Carreras B, Hitchings MDT, Yang B, Katzelnick LC, Rattigan SM, et al. A systematic review of antibody mediated immunity to coronaviruses: kinetics, correlates of protection, and association with severity. Nat Commun. 2020;11:4704. doi: 10.1038/s41467-020-18450-4 32943637 PMC7499300

[49] Juno JA, Tan HX, Lee WS, Reynaldi A, Kelly HG, Wragg K, et al. Humoral and circulating follicular helper T cell responses in recovered patients with COVID-19. Nat Med. 2020;26:1428–34. doi: 10.1038/s41591-020-0995-0 32661393

[50] Charlson ME, Pompei P, Ales KL, CR MK. A new method of classifying prognostic comorbidity in longitudinal studies: development and validation. J Chronic Dis. 1987;40:373–83. doi: 10.1016/0021-9681(87)90171-8 3558716

[51] Chi X, Yan R, Zhang J, Zhang G, Zhang Y, Hao M, et al. A neutralizing human antibody binds to the N-terminal domain of the Spike protein of SARS-CoV-2. Science. 2020;369:650–5. doi: 10.1126/science.abc6952 32571838 PMC7319273

[52] Zeng W, Liu G, Ma H, Zhao D, Yang Y, Liu M, et al. Biochemical characterization of SARS-CoV-2 nucleocapsid protein. Biochem Biophys Res Commun. 2020;527:618–23. doi: 10.1016/j.bbrc.2020.04.136 32416961 PMC7190499

[53] Shi SQ, Peng JP, Li YC, Qin C, Liang GD, Xu L, et al. The expression of membrane protein augments the specific responses induced by SARS-CoV nucleocapsid DNA immunization. Mol Immunol. 2006;43:1791–8. doi: 10.1016/j.molimm.2005.11.005 16423399 PMC7112551

[54] Shrock E, Fujimura E, Kula T, Timms RT, Lee IH, Leng Y, et al. Viral epitope profiling of COVID-19 patients reveals cross-reactivity and correlates of severity. Science. 2020. doi: 10.1126/science.abd4250 32994364 PMC7857405

[55] Mishra N, Huang X, Joshi S, Guo C, Ng J, Thakkar R, et al. Immunoreactive peptide maps of SARS-CoV-2. Commun Biol. 2021;4:225. doi: 10.1038/s42003-021-01743-9 33580175 PMC7881038

[56] Chen S, Zheng X, Zhu J, Ding R, Jin Y, Zhang W, et al. Extended ORF8 Gene Region Is Valuable in the Epidemiological Investigation of Severe Acute Respiratory Syndrome-Similar Coronavirus. J Infect Dis. 2020;222:223–33. doi: 10.1093/infdis/jiaa278 32433742 PMC7313917

[57] Zhang Y, Zhang J, Chen Y, Luo B, Yuan Y, Huang F, et al. The ORF8 Protein of SARS-CoV-2 Mediates Immune Evasion through Potently Downregulating MHC-I. bioRxiv. 2020; https://www.biorxiv.org/content/10.1101/2020.05.24.111823v1.

[58] Yoshimoto FK. The Proteins of Severe Acute Respiratory Syndrome Coronavirus-2 (SARS CoV-2 or n-COV19), the Cause of COVID-19. Protein J. 2020;39:198–216. doi: 10.1007/s10930-020-09901-4 32447571 PMC7245191

[59] Li JY, Liao CH, Wang Q, Tan YJ, Luo R, Qiu Y, et al. The ORF6, ORF8 and nucleocapsid proteins of SARS-CoV-2 inhibit type I interferon signaling pathway. Virus Res. 2020;286:198074. doi: 10.1016/j.virusres.2020.198074 32589897 PMC7309931

[60] Young BE, Fong SW, Chan YH, Mak TM, Ang LW, Anderson DE, et al. Effects of a major deletion in the SARS-CoV-2 genome on the severity of infection and the inflammatory response: an observational cohort study. Lancet. 2020;396:603–11. doi: 10.1016/S0140-6736(20)31757-8 32822564 PMC7434477

[61] Wang X, Lam JY, Wong WM, Yuen CK, Cai JP, Au SW, et al. Accurate Diagnosis of COVID-19 by a Novel Immunogenic Secreted SARS-CoV-2 orf8 Protein. MBio. 2020;11:e02431–20. doi: 10.1128/mBio.02431-20 33082264 PMC7587431

[62] Ren Y, Shu T, Wu D, Mu J, Wang C, Huang M, et al. The ORF3a protein of SARS-CoV-2 induces apoptosis in cells. Cell Mol Immunol. 2020;17:881–3. doi: 10.1038/s41423-020-0485-9 32555321 PMC7301057

[63] Lei X, Dong X, Ma R, Wang W, Xiao X, Tian Z, et al. Activation and evasion of type I interferon responses by SARS-CoV-2. Nat Commun. 2020;11:3810. doi: 10.1038/s41467-020-17665-9 32733001 PMC7392898

[64] Yuen CK, Lam JY, Wong WM, Mak LF, Wang X, Chu H, et al. SARS-CoV-2 nsp13, nsp14, nsp15 and orf6 function as potent interferon antagonists. Emerg Microbes Infect. 2020;9:1418–28. doi: 10.1080/22221751.2020.1780953 32529952 PMC7473193

[65] Galloway SE, Paul P, MacCannell DR, Johansson MA, Brooks JT, MacNeil A, et al. Emergence of SARS-CoV-2 B.1.1.7 Lineage—United States, December 29, 2020-January 12, 2021. MMWR Morb Mortal Wkly Rep. 2021;70:95–9. doi: 10.15585/mmwr.mm7003e2 33476315 PMC7821772

[66] Centers for Disease Control and Prevention. SARS-CoV-2 Variant Classifications and Definitions. 2021.

[67] Outbreak.info. SARS-CoV-2 (hCoV-19) Mutation Reports—Lineage Mutation Tracker. 2021; https://outbreak.info/situation-reports.

[68] ARTIC Network. Preliminary genomic characterisation of an emergent SARS-CoV-2 lineage in the UK defined by a novel set of spike mutations. 2020; https://virological.org/t/preliminary-genomic.

[69] Weisblum Y, Schmidt F, Zhang F, DaSilva J, Poston D, Lorenzi JC, et al. Escape from neutralizing antibodies by SARS-CoV-2 spike protein variants. Elife. 2020;9:e61312. doi: 10.7554/eLife.61312 33112236 PMC7723407

[70] Eguia R, Crawford KHD, Stevens-Ayers T, Kelnhofer-Millevolte L, Greninger AL, Englund JA, et al. A human coronavirus evolves antigenically to escape antibody immunity. bioRxiv. 2020; https://www.biorxiv.org/content/10.1101/2020.12.17.423313v1. 10.1371/journal.ppat.1009453 PMC8031418 33831132

[71] Woo PC, Lau SK, Huang Y, Yuen KY. Coronavirus diversity, phylogeny and interspecies jumping. Exp Biol Med (Maywood). 2009;234:1117–27. doi: 10.3181/0903-MR-94 19546349

[72] Anthony SJ, Johnson CK, Greig DJ, Kramer S, Che X, Wells H, et al. Global patterns in coronavirus diversity. Virus Evol. 2017;3:vex012. doi: 10.1093/ve/vex012 28630747 PMC5467638

[73] Heffron AS, Mohr EL, Baker D, Haj AK, Buechler CR, Bailey A, et al. Antibody responses to Zika virus proteins in pregnant and non-pregnant macaques. PLoS Negl Trop Dis. 2018;12:e0006903. doi: 10.1371/journal.pntd.0006903 30481182 PMC6286021

[74] Liu L, Wang P, Nair MS, Yu J, Rapp M, Wang Q, et al. Potent neutralizing antibodies against multiple epitopes on SARS-CoV-2 spike. Nature. 2020;584:450–6. doi: 10.1038/s41586-020-2571-7 32698192

[75] Yuan M, Wu NC, Zhu X, Lee CD, RTY S, Lv H, et al. A highly conserved cryptic epitope in the receptor binding domains of SARS-CoV-2 and SARS-CoV. Science. 2020;368:630–3. doi: 10.1126/science.abb7269 32245784 PMC7164391

[76] Wu Y, Wang F, Shen C, Peng W, Li D, Zhao C, et al. A noncompeting pair of human neutralizing antibodies block COVID-19 virus binding to its receptor ACE2. Science. 2020;368:1274–8. doi: 10.1126/science.abc2241 32404477 PMC7223722

[77] Moore JB, June CH. Cytokine release syndrome in severe COVID-19. Science. 2020;368:473–4. doi: 10.1126/science.abb8925 32303591

[78] Holmes CL, Peyton CG, Bier AM, Donlon TZ, Osman F, Bartels CM, et al. Reduced IgG titers against pertussis in rheumatoid arthritis: Evidence for a citrulline-biased immune response and medication effects. PLoS ONE. 2019;14:e0217221. doi: 10.1371/journal.pone.0217221 31136605 PMC6538243

[79] Matsuyama S, Nao N, Shirato K, Kawase M, Saito S, Takayama I, et al. Enhanced isolation of SARS-CoV-2 by TMPRSS2-expressing cells. Proc Natl Acad Sci U S A. 2020;117:7001–3. doi: 10.1073/pnas.2002589117 32165541 PMC7132130

[80] Webb B, Sali A. Comparative Protein Structure Modeling Using MODELLER. Curr Protoc Bioinformatics. 2016;54:5.6.1–5.6.37. 27322406 10.1002/cpbi.3 PMC5031415

[81] Emsley P, Lohkamp B, Scott WG, Cowtan K. Features and development of Coot. Acta Crystallogr D Biol Crystallogr. 2010;66:486–501. doi: 10.1107/S0907444910007493 20383002 PMC2852313

[82] Wilke CO. gridtext: Improved Text Rendering Support for ‘Grid’ Graphics. R package version 0.1.1. 2020.

[83] Gu Z, Eils R, Schlesner M. Complex heatmaps reveal patterns and correlations in multidimensional genomic data. Bioinformatics. 2016;32:2847–9. doi: 10.1093/bioinformatics/btw313 27207943

[84] Edgar. MUSCLE: multiple sequence alignment with high accuracy and high throughput. Nucleic Acids Res. 2004;32:1792–7. doi: 10.1093/nar/gkh340 15034147 PMC390337

