# Supplementary figures and images for: The landscape of antibody binding in SARS-CoV-2 infection
Source: PLoS Biol. 2021 Jun 18;19(6):e3001265. doi: 10.1371/journal.pbio.3001265 (PMC8245122; doi:10.1371/journal.pbio.3001265)

807-S-26 alignment

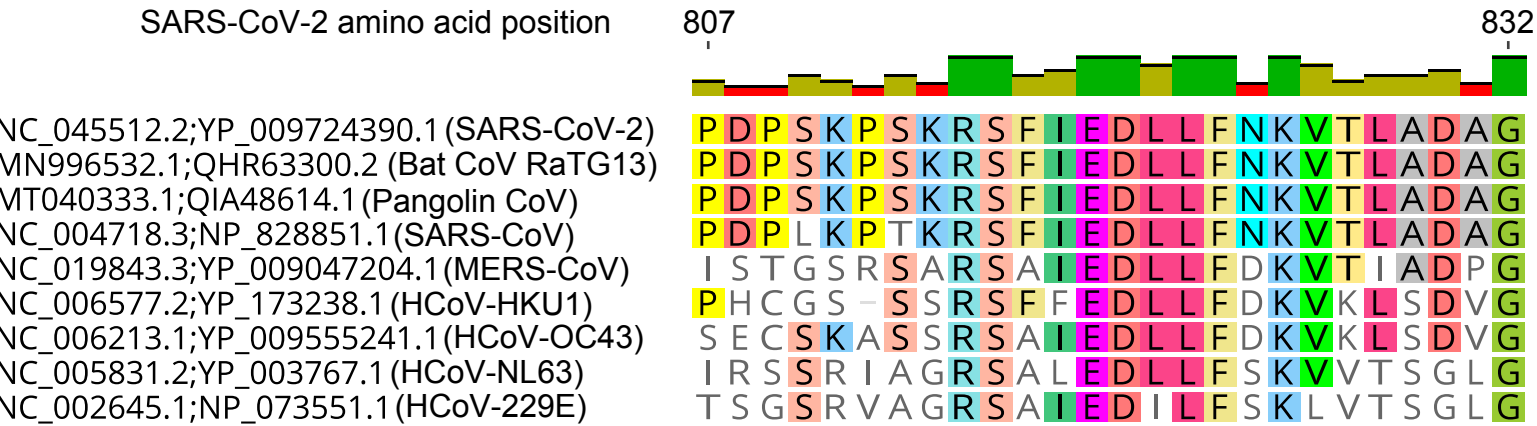

1140-S-25 alignment

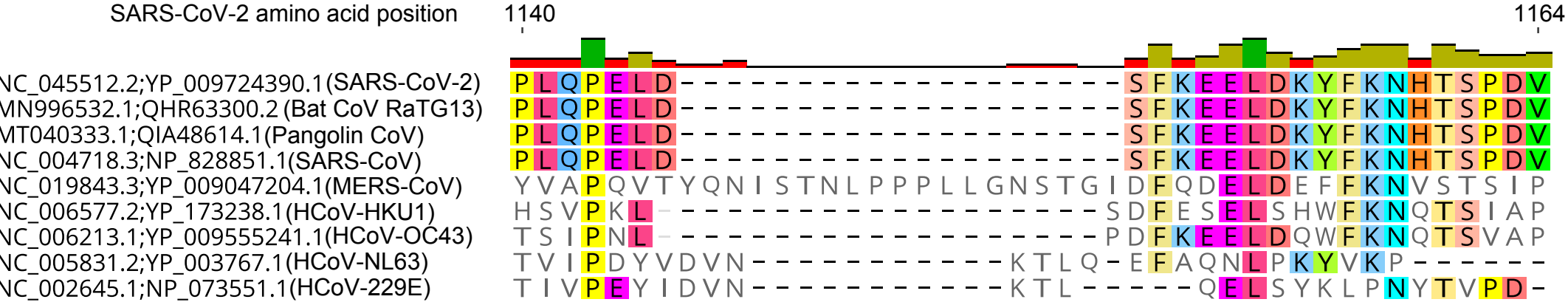

Supplement: S2 Fig — Alignments were performed in Geneious Prime 2020.1.2 (Auckland, New Zealand). CoV, coronavirus; COVID-19, coronavirus disease 2019. (PDF) [file pbio.3001265.s002.pdf]
