# Supplementary material for: The landscape of antibody binding in SARS-CoV-2 infection
Source: PLoS Biol. 2021 Jun 18;19(6):e3001265. doi: 10.1371/journal.pbio.3001265 (PMC8245122; doi:10.1371/journal.pbio.3001265)
Supplement: S5 Data — Supporting information Table A contains the proteins represented on the array, including the GenBank accession numbers and the number of replicates of reach peptide in those proteins. Supporting information Table B contains the characteristics of the 40 COVID-19 convalescent patients and the 20 naïve controls whose sera were used in this study. Supporting information Table C contains the characteristics of the 40 COVID-19 convalescent patients according to hospitalization status. COVID-19, coronavirus disease 2019. (DOCX) [file pbio.3001265.s012.docx]

**Supplementary Tables from Methods**

**Supplementary Table A. Proteins represented on the peptide microarray**

|  | **Protein(s)** | **GenBank accession number(s)** | **Number of replicates of each unique peptide** |
| --- | --- | --- | --- |
| **Coronavirus proteins** | Severe acute respiratory syndrome coronavirus 2 proteome | NC_045512.2 | 4-5 |
|  | Severe acute respiratory syndrome coronavirus proteome | NC_004718.3 | 3 |
|  | Middle Eastern respiratory syndrome coronavirus proteome | NC_019843.3 | 3 |
|  | Human coronavirus HKU1 proteome | NC_006577.2 | 3 |
|  | Human coronavirus OC43 proteome | NC_006213.1 | 3 |
|  | Human coronavirus 229E proteome | NC_002645.1 | 3 |
|  | Human coronavirus NL63 proteome | NC_005831.2 | 3 |
|  | Bat coronavirus (RaTG13 isolate) proteome | MN996532.1 | 3 |
|  | Pangolin coronavirus proteome | MT072864.1 | 3 |
| **Control proteins** | Human rhinovirus A1 polyprotein | NC_038311.1 | 3 |
|  | Human rhinovirus A7 polyprotein | DQ473503.1 | 3 |
|  | Human rhinovirus A16 polyprotein | L24917.1 | 3 |
|  | Human rhinovirus A36 polyprotein | JX074050.1 | 3 |
|  | Human rhinovirus C2 polyprotein | EF077280.1 | 3 |
|  | Human rhinovirus C15 polyprotein | GU219984.1 | 3 |
|  | Human rhinovirus C41 polyprotein | KY189321.1 | 3 |
|  | Human poliovirus 1 polyprotein | ANA67904.1 | 3 |

**Supplementary Table B. Characteristics of COVID-19 Convalescent and Control Subjects**

|  | COVID-19  (n=40) | Control  (n=20) | *p* |
| --- | --- | --- | --- |
| Age, median (IQR) years | 54 (34, 65) | 58 (41, 70) | 0.312 |
| Sex, number female (%) | 17 (42.5) | 11 (55.0) | 0.360 |
| Race, number (%) |  |  | 0.866 |
| White | 34 (85.0) | 18 (90.0) |  |
| Black | 3 (7.5) | 1 (5.0) |  |
| Asian | 3 (7.5) | 1 (5.0) |  |
| Native American | 0 (0.0) | 0 (0.0) |  |
| Pacific Islander | 0 (0.0) | 0 (0.0) |  |
| Ethnicity, number Hispanic (%) | 5 (12.5) | 1 (5.0) | 0.361 |
| Charlson comorbidity score, median (IQR) | 2 (0, 3) | 2 (0.5, 4) | 0.572 |
| Immunocompromised, number (%) | 9 (22.5) | 7 (35.0) | 0.302 |
| COVID-19 disease severity, number (%) |  |  |  |
| Hospitalized and intubated | 8 (20.0) | - | - |
| Hospitalized without intubation | 7 (17.5) | - | - |
| Not hospitalized | 25 (62.5) | - | - |

**Supplementary Table C. Characteristics of COVID-19 Convalescent Subjects According to Hospitalization Status**

| Characteristic | Not hospitalized (n=25) | Hospitalized without intubation (n=7) | Hospitalized and intubated (n=8) | *p* |
| --- | --- | --- | --- | --- |
| Age, median (IQR) years | 49 (30, 56) | 66 (48, 83) | 63 (58, 68) | 0.013 |
| Sex, number female (%) | 12 (48.0) | 3 (42.9) | 2 (25.0) | 0.519 |
| Race, number (%) |  |  |  | 0.537 |
| White | 20 (80.0) | 6 (85.7) | 8 (100.0) |  |
| Black | 3 (12.0) | 0 (0.0) | 0 (0.0) |  |
| Asian | 2 (8.0) | 1 (14.3) | 0 (0.0) |  |
| Native American | 0 (0.0) | 0 (0.0) | 0 (0.0) |  |
| Pacific Islander | 0 (0.0) | 0 (0.0) | 0 (0.0) |  |
| Ethnicity, number Hispanic (%) | 5 (20.0) | 0 (0.0) | 0 (0.0) | 0.180 |
| Charlson comorbidity score, median (IQR) | 1 (0, 2) | 2 (0, 6) | 2.5 (2, 4) | 0.082 |
| Immunocompromised, number (%) | 5 (20.0) | 2 (28.6) | 2 (25.0) | 0.875 |
