# Supplementary material for: The landscape of antibody binding in SARS-CoV-2 infection
Source: PLoS Biol. 2021 Jun 18;19(6):e3001265. doi: 10.1371/journal.pbio.3001265 (PMC8245122; doi:10.1371/journal.pbio.3001265)
Supplement: Attachment — Submitted filename: Ab Landscape in SARS-CoV-2 infection_ReviewerResponse_04.26.pdf [file pbio.3001265.s013.pdf]

Response to reviewer comments for “The landscape of antibody binding in SARS-CoV-2 infection”, PBIOLOGY-D-21-00032R1

*We thank the reviewers for their thoughtful and generally positive reviews. Below we provide a point-by-point response to each of their concerns (**bolded text** immediately following the reviewers’ requests), and point out where within the manuscript relevant changes have been made. All data not included in the Supporting Information files can be found at: https://github.com/Ong-Research/UW_Adult_Covid-19, and we have noted this in the legend for each figure, as requested by the Editor. Also, please note that this version of the manuscript does not have the figures included in the text; we removed the in-text figures in order to keep each file under 10MB in size, at the request of the Publications Assistant. Each figure is still available at the end of the manuscript, and the figure legends remain within the text.*

Reviewer #1

In this manuscript, Heffron et al. report linear peptide binding antibodies from pre-pandemic and SARS-CoV-2 infected patient serum samples to the S, M, N, ORF1ab, ORF3a, ORF6, and ORF8 proteins of SARS-CoV-2 as well as to proteins in the common cold CoVs HKU1, OC43, NL63, and 229E. The extent of this binding was measured first by peptide microarray and then validated by ELISA with selected peptides of interest. Several studies to date have performed similar types of analyses characterizing linear epitopes recognized by convalescent SARS-CoV-2 sera to SARS-CoV-2 proteins as well as other CCCoV proteins thus this limits the overall impact of the present study. Furthermore, the rather cursory and brief analysis of the data (in both the results section and discussion) does disservice to the work itself and ultimately fails to convey to the reader the overall importance.

The manuscript would be strengthened if the authors could comment on:
- Whether the non-homologous, non-identical epitopes recognized by SARS-CoV-2 positive sera in RaTG13, pangolin CoV, and SARS-CoV are conserved within themselves and comment on what potentially may be inducing these specific responses.
We agree with the reviewer that the antibody responses to these epitopes were intriguing and that it would benefit readers to include more in-depth discussion of these responses. We also realize that the way we described these epitopes may have been confusing, so we have clarified our wording and expanded our description of them in the Results (“Anti-SARS-CoV-2 antibodies may cross-reactively bind peptides in other CoVs” section of the Results), where we now describe these epitopes as “epitopes without a homologous SARS-CoV-2 epitope.” We have added additional columns to clarify our meaning in Supporting information S8 Data (columns identifying whether the epitope was in a region identical with SARS-CoV-2, was in a region non-identical but homologous with a region of SARS-CoV-2 which was an epitope, was in a region that did not have an epitope in the homologous region of SARS-CoV-2, or was in a region with no homologous region at all in SARS-CoV-2). We have also expanded and clarified our description of these epitopes in the Results (“Anti-SARS-CoV-2 antibodies may cross-reactively bind peptides in other CoVs” section of the Results), and we have expanded our consideration of these epitopes in the Discussion (fifth paragraph of the Discussion),

commenting upon the consideration that these epitopes may reflect a generalized immune activation.

- The authors mention that sera can recognize B1.1.7 ORF8 and N binding—is that notable—as aren't these regions conserved between B1.1.7 and SARS-CoV-2? Additionally, it would be more informative and noteworthy to assess whether sera recognized variant/mutated versions of the S peptides for the B1.1.7, P.1, and B.1.351 variants.
We agree with the reviewer that the results regarding variants of concern are quite interesting. B.1.1.7 has a number of mutations in ORF8 and nucleocapsid, compared to the original Wuhan virus (https://www.cdc.gov/mmwr/volumes/70/wr/mm7003e2.htm). The interesting finding from our data was that many of the antibodies we detected bound to areas that had mutated in B.1.1.7 compared to the original virus. We have revised our language to clarify that finding (see the fourth paragraph of the Discussion). Less was known about the P.1 and B.1.351 variants when we originally submitted this manuscript, but now that more data on them is available, we are happy to be able to include an analysis of them and of several other variants of concern the CDC has very recently named (https://www.cdc.gov/coronavirus/2019-ncov/cases-updates/variant-surveillance/variant-info.html) in this revision (see S11 Data and the fourth paragraph of the Discussion). We had not included the variants of concern on this array when we conducted these experiments, since the variants of concern had not yet been identified at that point, but we agree with the reviewer that it will be very interesting to see whether and how well sera may recognize peptides from the variants of concern tiled on the array, and we have added language about this, as a future direction, to the Discussion (fourth paragraph of the Discussion).

- The observation that 9 epitopes appeared to correlate with disease severity is interesting. While the data is presented in Figure 7 a more thorough description of these epitopes would be informative. Additionally, were any epitopes found that correlated with decreased disease severity?
We agree with the reviewer that these findings were intriguing. We have added a more thorough description of these epitopes, as requested (see the "Reactivity in some epitopes correlates with disease severity" of the Results). Using multilinear regression accounting for age, sex, immunocompromising conditions, and Charlson comorbidity index score to determine epitope-level resolution of differences in reactivity (see the "Reactivity in some epitopes correlates with disease severity" section of the Results and the "Statistical analysis" section of the Methods), no epitopes correlated with decreased disease severity. This data was previously included in Extended data 8 (now S10 Data) we have now added a description of it to the "Reactivity in some epitopes correlates with disease severity" section of the Results.

- In the Discussion, the authors comment "The ACE2 binding site and S-helix in extended fusion are not as immunodominant as expected suggesting that other, less-investigated may be playing a larger role in immunity to SARS-CoV-2…" is problematic. Arguably, the vast majority of the elicited response to vaccination or infection is conformational specific. Using linear peptides, as done in this microarray, will not "capture" these responses. For the authors to suggest that ACE2 binding site, which is, in fact, a conformational specific epitope recognized, predominantly by conformational specific antibodies (e.g., B38, ADI-) is not "immunodominant" in their assay is misleading and inaccurate. Inclusion of RBD and/or Spike proteins (not peptides derived from them) for comparison on the microarray are necessary controls. Furthermore, it is unclear what the authors mean by "S-helix in extended fusion".

We agree with the reviewer that many important epitopes in SARS-CoV-2 are likely conformational, and we have highlighted this point in the text (fifth paragraph of the Discussion). Nonetheless, a number of studies have found neutralizing antibodies produced against multiple different linear epitopes (see, for example, PMIDs 32483236, 32612199, and 32895485). We have clarified the phrasing that the reviewer pointed out, so that it further emphasizes that our findings are suggestive of the importance of non-RBD, non-conformational epitopes in SARS-CoV-2 in addition to critically important conformational epitopes and epitopes in the RBD (fifth paragraph of the Discussion), and we have changed the word "immunodominant" to "reactive, by these methods (second paragraph of the Discussion). While we agree with the reviewer that it would be interesting to compare results from our microarray work with conformational epitopes, RBD, and whole spike proteins, we also agree with the editor that such experiments would be beyond the scope of this manuscript. By "S helix in extended fusion" we had intended to communicate that the RBD in general was not as reactive as expected. We are grateful to the reviewer for noticing this language, which we agree is confusing. We have updated the text to reflect our intentions about the RBD (second paragraph of the Discussion).

Minor:
- Discussion paragraph 2: "though that may be due to using an earlier sample" is ambiguous phrasing leaving it unclear which sample (anti-M positive or negative) is earlier.
We appreciate the reviewer's desire for clarity. We have increased the description provided in the paragraph as requested (see the second paragraph of the Discussion).

- Discussion paragraph 4: "especially given that pre-existing anti-CoV antibodies are more common in children and adolescents" implies a connection between protection and age that is not clearly stated in the text and should be clarified.
We sincerely appreciate the reviewer's attention to detail. Upon discussion among our authorship team, we decided this statement was less related to the current analyses and somewhat speculative, and thus we have removed the sentence.

- In the Acknowledgements what is meant by "chimeric PDB file" used for Figure 4
We appreciate the reviewer's thoroughness. We have increased our description of these methods in the Methods and the Acknowledgments.

Figures
- Figures 2 and 3: the black arrows are not defined in the figure legends.
We are not certain about what the reviewer is requesting for Figure 2, since there are no black arrows in Figure 2. There are black diamonds in Figure 2, with a description included in Figure 2 itself; we have added a description of the diamonds to the figure legend of Figure 2. We appreciate the reviewer catching our oversight on Figure 3; we have added a description of the black arrows (and the black stars) to the Figure 3 legend. We are happy to adjust Figure 2 as necessary if given additional guidance.

- Figure 4: a scale bar for the heatmap should be included.
We appreciate the reviewer's attention to detail, and we have added a scale bar for the structures in Figure 4.

- Figure 4: use of a rotated arrow versus a linear one that denotes a 45deg rotation should be used

We have changed the arrows from a linear shape to a curved shape in Figure 4.

- Figure 4: the authors use the "one up RBD" structure; it would be useful to readers who are not familiar with the S structure to include a non-heat map version that labels key regions (e.g., RBD, RBM, fusion peptide, "base").
We appreciate the reviewer's attention to detail. We have added a version of the structure with key regions labeled as requested (see Figure 4D).

Reviewer #2

In this manuscript, Heffron and colleagues designed a peptide microarray of SARS-CoV-2 and other coronaviruses to assess antibody epitope specificity and potential cross-reactivity with other CoVs in COVID-19 convalescent patients and demonstrated previously unknown, highly reactive B cell epitopes throughout the full proteome of SARS-CoV-2 and other CoV proteins. An epitope in the N-terminus of M protein with high specificity and sensitivity to the serum of the COVID-19 patients was found. This study will be useful for vaccine design and serological diagnosis. This reviewer just has a few concerns.

1. Humoral immunity to virus is related to infection time. It suggested that information of sample collecting time post disease onset should be included somewhere in the manuscript.
We agree with the reviewer that inclusion of the date of disease onset might be useful to readers. Unfortunately, we did not collect the date of disease or symptom onset from our study subjects. We did, however, collect the date of the first positive PCR test, which we have included in the manuscript (Supporting information S3 Data), and we have commented on the collection of symptom onset data and its correlation with antibody binding as a future direction in the Discussion (sixth paragraph of the Discussion section).

2. Neutralizing titers of the sera from COVID-19 patients and the controls should be provided. The control sera showed cross reactivity with the SARS-CoV-2 peptide microarray. Did the control sera neutralize SARS-CoV-2?
We thank the reviewer for this insight, and we agree that neutralization titers would be potentially useful data. We included neutralization titers from all patients in Supporting information S3 Data. As part of a larger study, neutralizing titers were performed for 30 SARS-CoV-2-naïve control subjects' samples collected prior to 2019, including 12 out of our 20 SARS-CoV-2-naïve controls. None of these showed neutralizing activity (Supporting information S3 Data), and given time, resource, and sample amount constraints, we did not perform neutralizing assays on the remaining 8 controls. We have included additional discussion of this data in lines the Results ("SARS-CoV-2-naïve controls show consistent binding in "common cold" CoVs and limited binding in SARS-CoV-2, SARS-CoV, and MERS-CoV" and "SARS-CoV-2 infection induces antibodies binding throughout the proteome" sections of the Results) and the Discussion (second paragraph of the Discussion).

3. It will be of greater interest to determine whether epitopes identified in this study could elicit neutralizing antibodies after vaccination.

We agree with the reviewer, and we look forward to seeing or contributing to future studies investigating the vaccine potential of the epitopes we described. We discuss this future direction in the second and third paragraphs of the Discussion.
